# IMU- and Vision-Based Measurement Techniques for Joint Kinematics: A Narrative Review

**DOI:** 10.3390/s26165063

**Published:** 2026-08-10

**Authors:** Luca Ceriola, Luca Molinaro, Juri Taborri, Fabrizio Patanè, Ilaria Mileti

**Affiliations:** 1 Department of Engineering, University Niccolò Cusano, 00166 Rome, Italy; fabrizio.patane@unicusano.it; 2 Department of Theoretical and Applied Sciences (DiSTA), eCampus University, 22060 Novedrate, Italy; luca.molinaro@uniecampus.it; 3 Department of Economics Engineering Society and Business Organization (DEIM), University of Tuscia, 01100 Viterbo, Italy; juri.taborri@unitus.it

**Keywords:** joint kinematics, inertial measurement units, markerless motion capture, computer vision, biomechanical validation, measurement accuracy, sensor fusion, biomechanics, gait analysis

## Abstract

Accurate assessment of joint kinematics is fundamental to biomechanics, rehabilitation, and sports science. Although optical motion capture (OMC) remains the laboratory reference standard for biomechanical validation, its cost, infrastructure requirements, and limited applicability outside controlled environments restrict its broader use. Wearable inertial measurement units (IMUs) and vision-based markerless systems have consequently emerged as complementary alternatives, offering portability, reduced subject preparation, and applicability in ecological settings. Their rapid development, however, has not always been accompanied by an equally rigorous metrological interpretation of performance. This narrative review provides a comparative analysis of IMU- and vision-based approaches for joint kinematics estimation, focusing on biomechanical validation metrics and measurement error. Because the primary literature reports fundamentally different quantities under widely differing experimental conditions, evidence is presented stratified by outcome class, joint, plane of motion, task, and acquisition dimensionality, and values belonging to different outcome classes are not pooled. For sagittal-plane lower-limb angles during level walking in healthy adults, with careful sensor-to-segment calibration and an optoelectronic reference, IMU-based systems show the most consistent performance, with RMSE commonly between 3° and 6°. Vision-based systems achieve comparable accuracy for selected outcomes, particularly spatiotemporal gait parameters and sagittal-plane angles in controlled views, while degrading with occlusion, motion blur, and depth ambiguity. Accuracy is therefore not an intrinsic property of the sensing modality but of the entire measurement chain, including calibration, biomechanical modeling, acquisition geometry, and reporting conventions. Rather than ranking technologies by accuracy alone, the measurement requirements should be derived from the intended application.

## 1. Introduction

Accurate evaluation of joint kinematics is fundamental to biomechanics, rehabilitation, ergonomics, and sports science, because clinically meaningful interpretations often depend on relatively small differences in joint-angle trajectories, range of motion, coordination patterns, and inter-limb asymmetries. In validation studies, marker-based optoelectronic motion capture (OMC) is commonly adopted as the laboratory reference standard for benchmarking alternative measurement systems [1]. However, OMC-derived kinematics are not error-free. Camera calibration, reconstruction uncertainty, marker placement, filtering choices, and missing-marker handling can all affect reconstructed trajectories and propagate to segment pose and joint-angle estimates [1,2]. Therefore, for emerging technologies, the key issue is not simply whether they “match” OMC but under which biomechanical definitions, acquisition protocols, and modeling assumptions agreement can be expected, reproduced, and interpreted in a clinically meaningful way.

Among the technologies that have most expanded access to human movement analysis, two families have emerged as particularly relevant: wearable inertial measurement units (IMUs) and vision-based markerless systems. IMU-based systems are attractive because they are portable, relatively low-cost, and deployable outside dedicated laboratories, thus enabling movement assessment in clinics, homes, workplaces, sports environments, and other real-world settings. Their growing adoption is supported by a broad body of literature showing that their validity and reliability depend strongly on the joint under investigation, the plane of motion, the attachment procedure, and the experimental protocol [3,4,5,6,7,8]. At the same time, IMU-derived joint kinematics are intrinsically indirect: joint angles are inferred through a multistep pipeline that includes orientation estimation, sensor-to-segment alignment, and biomechanical modeling, often with inverse-kinematics constraints. Across this chain, sensor-to-segment calibration remains a major source of systematic offsets and range-of-motion distortions, and it is one of the main reasons why results are often difficult to compare across studies [9,10,11].

Vision-based markerless systems follow a fundamentally different logic. Rather than inferring movement from body-worn sensors, they estimate human pose directly from video without physical markers. Recent progress in deep learning has substantially improved both 2D and 3D pose estimation, enabling markerless workflows ranging from monocular video analysis to multi-view 3D reconstruction and downstream musculoskeletal modeling [12,13,14,15]. These methods reduce subject preparation time, provide non-contact measurement, and can be deployed rapidly even in constrained environments. Nevertheless, their accuracy remains highly dependent on acquisition and scene conditions, including viewpoint, image resolution, lighting, motion blur, occlusion, and the strategy used to convert image-derived keypoints into anatomically meaningful segment frames and joint angles [12,15,16].

A substantial number of reviews are already available for both technology families. On the IMU side, previous works have mainly focused on gait validity and reliability, sensor-to-segment calibration, or specific anatomical regions such as the upper limb. On the vision side, most reviews have concentrated on 2D/3D pose-estimation algorithms, markerless motion-capture pipelines, or application-specific domains such as gait, rehabilitation, or sports. These contributions are highly valuable, but they are often modality-specific, application-specific, or primarily algorithm-oriented. As a result, the literature still lacks a sufficiently integrated discussion of how IMU- and vision-based systems compare when examined within the same metrological framework, namely in terms of joint definitions, modeling assumptions, validation metrics, and dominant sources of measurement error [12].

This gap is increasingly relevant because IMU- and markerless systems are now used in partially overlapping scenarios, including rehabilitation monitoring, sports performance analysis, ergonomics, and out-of-laboratory assessment. In such contexts, their outputs are often discussed side by side, despite the fact that the two technologies differ substantially in observability, processing pipelines, and error mechanisms. A comparative review is therefore needed not only to summarize representative accuracy values but also to clarify what those values actually mean, how they were obtained, and under which conditions they can be considered comparable and clinically interpretable.

Accordingly, this review provides a comparative analysis of IMU- and vision-based approaches for estimating joint kinematics, with particular emphasis on evaluation metrics, measurement errors, and methodological dependencies. Specifically, the review (i) summarizes the most commonly used pipelines for both technologies; (ii) discusses how joint kinematics are defined within a biomechanical framework; (iii) compares representative validation results across joints, planes of motion, and tasks; and (iv) highlights the main factors that limit cross-study comparability, including calibration procedures, coordinate-system definitions, biomechanical modeling choices, and reporting conventions. By framing IMU and markerless methods within a common validation perspective, this review aims to support a more informed interpretation of the literature and a more reasoned selection of motion-analysis technologies in research and applied settings.

The remainder of the paper is organized as follows. Section 2 states the scope of the review and the criteria used to select the studies discussed. Section 3 summarizes the main related reviews and clarifies the specific gap addressed here. Section 4 recalls the biomechanical principles underlying joint kinematics and the role of standardized coordinate-system definitions. Section 5 and Section 6 examine, respectively, IMU-based and vision-based pipelines. Section 7 discusses the evaluation metrics most commonly used to assess performance, while Section 8 compares representative validation studies and measurement errors across technologies and sets out the remaining challenges—including the outlook for hybrid visual–inertial and data-driven solutions—alongside the evidence that bears on them. Section 9 states the main findings.

## 2. Scope of the Review and Selection of Studies

### 2.1. Type of Review and Rationale

This article is a narrative review, and in the writing process, the PRISMA framework was not adopted. The objective of this work is interpretive: its purpose is to clarify what reported accuracy values for IMU- and vision-based joint kinematics actually mean and under which definitions, protocols, and modeling assumptions they can be compared. Studies differ in the reference system adopted, in the anatomical coordinate system and joint-angle conventions applied, in the calibration procedure, in the task and population examined, and in the dimensionality of the acquisition (2D projection vs. 3D reconstruction). Given this degree of methodological heterogeneity, providing summary results without extensive contextual explanation would not be metrologically meaningful.

Several rigorous systematic reviews already exist for individual segments of this field, and they are discussed in Section 3. The contribution intended here is complementary: to place the two technology families within a single metrological frame of reference rather than to re-derive their pooled performance.

### 2.2. Identification of the Literature

The relevant literature was identified through searches of Scopus, Web of Science, PubMed, and IEEE Xplore, complemented by Google Scholar and by backward and forward citation tracking from the reviews discussed in Section 3. Searches combined terms describing the measurement technology (*inertial measurement unit*, *IMU*, *MIMU*, *wearable sensor*, *markerless*, *pose estimation*, *computer vision*, *video-based motion capture*) with terms describing the measurand and its evaluation (*joint kinematics*, *joint angle*, *gait analysis*, *validity*, *reliability*, *accuracy*, *validation*, *optoelectronic*, and *motion capture*). Only peer-reviewed, full-text English-language publications were considered, and no lower date limit was imposed.

Article selection was performed by the authors with respect to topic relevance and methodological informativeness.

### 2.3. Criteria for Studies Included in the Quantitative Comparison

For a study to be included in this narrative review, it had to meet these criteria:

1. Report an experimental comparison against a reference system, such as marker-based optoelectronic motion capture or an instrumented walkway for spatiotemporal gait parameters;
2. Report results using a named metric (e.g., RMSE, MAE, RMSD, mean difference, ICC, Pearson’s *r*), rather than qualitative or purely visual comparison;
3. Report sufficient and relevant results for a specific joint or segment, plane of motion or degree of freedom, and task.

Studies reporting only simulated or synthetically generated data, without an experimental reference measurement, were excluded from the quantitative comparison, although they are discussed where they illustrate methodological developments.

### 2.4. Data Extraction and Stratification

For each retained study, we recorded the following:

- Sensors used.
- Dimensionality of pose reconstruction (2D/3D).
- Reference system.
- Population and task.
- Joints, planes, or degrees of freedom evaluated.
- Evaluation metrics.

Due to the wide variety of metrics reported across these studies, the findings are categorized separately rather than being pooled. Four outcome classes are kept separate throughout: joint angles, joint center positions, spatiotemporal parameters, and classification performance. Joint-angle results are further subgrouped by joint, plane, task, and dimensionality. Therefore, any indicative range that we report refers specifically to one outcome class under stated conditions.

## 3. Related Works

This section explores the current state of IMU- and video- based methodologies, highlighting key reviews that address specific aspects of human kinematics measurement.

The methodological foundations of IMU-based kinematics measurement are a key focus in several reviews. Weygers et al. (2020) [17] conducted a systematic review addressing inertial sensor-based lower limb joint kinematics, with a focus on methodological requirements for clinical applicability and future research directions. Pacher et al. (2020) [9] systematically reviewed sensor-to-segment calibration methodologies critical for accurate lower-body kinematic analysis with inertial sensors. These reviews emphasize the importance of methodological rigor in IMU-based assessments to ensure clinical relevance and guide further research. Fang et al. (2023) [11] systematically reviewed the conversion of IMU data to joint angles, particularly in the upper limb, addressing a key challenge in applying IMUs to upper extremity motion analysis. Expanding beyond kinematics, Lee and Lee (2022) [18] presented a systematic review on estimating joint kinetics using inertial motion capture systems, highlighting the growing importance of kinetic analysis in addition to kinematics. Ghattas & Jarvis (2024) [19] provided a systematic review of the validity of IMUs for tracking human motion, offering a broad perspective on the accuracy of IMU-based measurements. Furthermore, Kobsar et al. (2020) [3] conducted a systematic review and meta-analysis on the validity and reliability of wearable inertial sensors in healthy adult walking, while Zeng et al. (2022) [20] focused on the validity and reliability of IMUs for measuring lower extremity kinematics during running, finding excellent agreement for spatiotemporal outcomes but considerably larger discrepancies for joint angles. Schall et al. (2022) [21] offered a brief overview of wearable inertial sensors for objective kinematic assessments, summarizing their utility in various settings.

IMU technology is also being applied in specific scenarios and populations. Kranzinger et al. (2023) [22] scoped the classification methods for human motion data using IMUs in sports, providing insights into how IMUs are used to analyze and classify athletic movements. Rana and Mittal (2021) [23] reviewed wearable sensors for real-time kinematics analysis in sports, focusing on how these sensors provide feedback for performance enhancement. Gu et al. (2023) [24] reviewed IMU-based motion capture systems within rehabilitation, exploring system designs, applications, and future developments. Prill et al. (2021) [25] systematically reviewed the diagnostic accuracy and clinical applications of wearable movement sensors specifically for knee joint rehabilitation. Weizman et al. (2022) [26] presented the recent state of wearable IMU sensor use in people living with spasticity, highlighting the clinical utility of IMUs in assessing and managing this condition. Zampogna et al. (2020) [27] reviewed fifteen years of wireless sensors for balance assessment in neurological disorders, demonstrating the utility of IMUs in clinical settings for evaluating patients with neurological conditions. Digo et al. (2022) [28] presented a narrative review on wearable inertial sensors for human motion tracking in industrial scenarios, focusing on the role of IMUs in enhancing human–robot interaction and worker safety. Salisu et al. (2023) [29] reviewed motion capture technologies for ergonomics, including IMUs, and their application in assessing and improving workplace conditions. Bo et al. (2022) [30] reviewed IMU-based monitoring for assistive diagnosis and management within the Internet of Health Things (IoHT), highlighting the potential of IMUs in remote health monitoring and management. Ashry et al. (2024) [31] reviewed transfer learning methods applied to human activity recognition using IMU data, demonstrating advanced analytical techniques.

On the other hand, the analysis of human motion and pose estimation through video-based systems has experienced considerable development in recent years, fostering a wide array of applications across various fields. A fundamental area of research for video-based systems is human activity recognition (HAR), which involves recognizing activities from a series of observations; this has been comprehensively explored in reviews by Ke et al. (2013) [32] and Zhang et al. (2019) [33], with Ke et al. (2013) [32] detailing the recognition of activities crucial for applications like video surveillance, healthcare, and human–computer interaction, and Zhang et al. (2019) [33] outlining the evolution of HAR approaches, progressing from global to local and depth-based representations, alongside classification methods, including template-based, discriminative, and generative models. Al-Faris et al. (2020) [34] also contributed to the understanding of human action recognition, reviewing computer-vision-based methodologies that span from hand-crafted representations to deep learning technologies.

Concurrent to these advancements, significant strides have been made in human pose estimation. Dang et al. (2019) [35] surveyed deep-learning-based 2D human pose estimation methods, underscoring the substantial impact of deep learning on enhancing pose estimation performance. Munea et al. (2020) [36] also presented a survey and taxonomy of models applied in 2D human pose estimation, which is a critical component for various tasks such as action recognition and human–computer interaction. Chen et al. (2022) [13] summarized recent achievements in 2D human pose estimation, focusing on network architecture design, training refinement, and post-processing techniques. Zheng et al. (2023) [14] reviewed deep-learning-based solutions for 2D and 3D pose estimation, analyzing and comparing methods based on input data and inference procedures, and addressing challenges like insufficient data and occlusions. Desmarais et al. (2021) [12] reviewed 3D human pose estimation algorithms for markerless motion capture, focusing on metrics, benchmarks and method structures, and proposed a taxonomy based on accuracy, speed, and robustness. Neupane et al. (2024) [37] contributed a survey on deep 3D human pose estimation, reviewing contemporary strategies including Convolutional Neural Networks, Graph Convolutional Networks, Transformers and their combinations.

Moreover, Colyer et al. (2018) [38] reviewed the broader evolution of vision-based motion analysis, tracing its progression from manual techniques to markerless systems, driven by the increasing demand for efficient and less intrusive methods of kinematic data collection. Table 1 arranges these prior reviews chronologically and by technology family, summarizing the key topic and main finding of each.

These developments have enabled video-based motion analysis to be applied in several distinct areas. For instance, Simor et al. (2016) [41] explored usability evaluation methods for gesture-based games, emphasizing the growing significance of gestural interaction systems in promoting physical and mental health. In sports, Mooney et al. (2015) [40] illustrated the application of video-based methods for competitive swimming analysis, highlighting the technology’s capacity to extract key performance metrics. Badiola-Bengoa and Mendez-Zorrilla (2021) [43] specifically reviewed camera-based human pose estimation in sports and exercise, demonstrating its increasing use in this field. Furthermore, Muro-de-la-Herran et al. (2014) [39] provided an overview of gait analysis methods, comparing wearable and non-wearable systems within clinical contexts, while Parks et al. (2019) [42] specifically assessed the potential of low-cost video-based motion analysis options for clinical rehabilitation by evaluating their validity and reliability against gold-standard 3D biomechanical methods. Amsaprabhaa et al. (2021) [44] surveyed spatio-temporal frameworks for kinematic gait analysis in RGB videos, focusing on spatio-temporal features and kinematic joint points. Vun et al. (2024) [48] reviewed vision-based motion capture for gait analysis in the context of neurodegenerative diseases, examining the potential of these systems for early detection and monitoring. Rybnikar et al. [46] (2022) reviewed the use of motion capture technology for ergonomics evaluation, addressing the risk of musculoskeletal disorders in manual activities. Sardari et al. (2023) [47] reviewed AI techniques for skeleton-based physical rehabilitation action evaluation, highlighting the role of vision-based sensors in home-based rehabilitation. Huamanchahua et al. (2022) [45] provided a review of human cinematic capture and movement systems using Kinect.

Collectively, these reviews highlight the continuous progress in IMU- and video-based motion analysis, its broadening scope of applications, and the persistent drive to create more advanced and practical systems.

## 4. General Principles and Recommendations for Human Joint Kinematics

### 4.1. Introduction

Human joint kinematics describes the relative motion between adjacent body segments. Within a general biomechanical framework, the instantaneous joint configuration can be represented by a position vector, which captures translation, and an orientation matrix, which captures rotation. Together, these quantities provide a complete six-degrees-of-freedom (6-DOF) description of intersegmental motion, comprising three translational and three rotational components.

For these measurements to be meaningful and reproducible, two methodological prerequisites must be met: (i) the definition of anatomical coordinate systems (ACSs) and (ii) the adoption of a consistent convention for decomposing joint orientation into clinically interpretable angles. Anatomical coordinate systems are defined from palpable bony landmarks so that segment-level reference frames can be replicated across subjects, sessions, and laboratories. Joint rotations are then typically expressed using a Cardan/Euler sequence, in which the orientation of the distal segment relative to the proximal one is decomposed into three ordered rotations, commonly associated with flexion/extension, abduction/adduction, and internal/external rotation.

However, different decomposition conventions, such as Cardan angles, geometric approaches, or projections of orientation vectors onto anatomical planes, can yield different numerical values for the same physical movement, especially for rotations outside the sagittal plane. This methodological variability has long been recognized as a major obstacle to cross-study comparability and clinical interpretation.

These conventions are fundamental for the technologies reviewed in this work. Both IMU-based and vision-based systems must map their native outputs onto anatomically defined coordinate systems before joint angles can be computed. In IMU-based pipelines, this mapping is achieved through sensor-to-segment (S2S) calibration, which estimates the rotation between the sensor’s technical frame and the anatomical frame of the underlying body segment [9,10]. In vision-based systems, image-derived keypoints must be converted into segment-level anatomical frames, often by means of marker augmentation models or musculoskeletal Inverse Kinematics [15,16]. In both cases, discrepancies in anatomical frame definitions are a major source of systematic error and inter-study disagreement [10].

To address these issues, the International Society of Biomechanics (ISB) has proposed standardized recommendations for reporting joint kinematics based on the joint coordinate system (JCS) introduced by Grood and Suntay (1983) [49]. Originally developed for the knee, the JCS framework was subsequently extended to other joints in two landmark publications: the first one covering the ankle, hip, and spine, and the second one, covering the shoulder, elbow, wrist, and hand. More recently, in response to the rapid expansion of wearable sensing, the ISB published dedicated recommendations for the definition, estimation, and reporting of joint kinematics obtained using inertial measurement technology, with explicit attention to coordinate-system definition, calibration, sensor fusion, and quality assessment in IMU-based workflows [10].

The following subsections summarize the ISB anatomical definitions for the major human joints. For each joint, we highlight the specific measurement challenges that arise when these definitions are implemented using IMU- or vision-based technologies, as these challenges directly influence the accuracy values reported in Section 5, Section 6, and Section 8. The selected joints were chosen according to two criteria: (i) their relevance to clinical and biomechanical practice, as they are among the joints most frequently analyzed in gait analysis, rehabilitation, sports science, and ergonomics, and (ii) the availability of formal ISB JCS definitions. The selection therefore includes the lower limb (ankle, knee, hip), upper limb (shoulder, elbow, wrist and hand), and trunk (spine). Other joints for which ISB definitions are available, such as the temporomandibular joint, were not included because they fall outside the primary scope of this review. Table 2 provides a synoptic overview of the ISB JCS definitions and the associated technology-specific measurement challenges.

### 4.2. Anatomical Definitions

#### 4.2.1. Ankle Joint Complex

The ankle joint complex comprises both the talocrural and subtalar joints. In the ISB framework, the coordinate system is defined from anatomical landmarks including the medial and lateral malleoli and tibial reference points. Joint motion is primarily described in terms of dorsiflexion/plantarflexion, inversion/eversion, and internal/external rotation.

##### Technology-Specific Challenges

The ankle is widely recognized as one of the most difficult joints to assess accurately with both IMU- and vision-based approaches. For IMU systems, the relatively small amplitudes of inversion/eversion and internal/external rotation, combined with soft-tissue artifacts over the foot and shank, make the estimates highly sensitive to S2S calibration errors. Lower-limb IMU studies consistently report that calibration choice materially affects accuracy and reproducibility, particularly outside the sagittal plane [50,51]. For vision-based systems, ankle estimation is further complicated by occlusion from footwear, the small size of distal foot segments relative to image resolution, and the limited robustness of distal lower-limb keypoint detection, especially in monocular or low-resolution settings [16,52].

#### 4.2.2. Knee Joint

Within the ISB JCS, the knee is modeled as a modified hinge joint, with flexion/extension as the primary rotational degree of freedom. The femoral coordinate system is defined using the medial and lateral epicondyles, whereas the tibial coordinate system relies on landmarks such as the tibial tuberosity and malleolar axis. In addition to flexion/extension, the JCS includes secondary rotations, varus/valgus and internal/external rotation, as well as translational components.

##### Technology-Specific Challenges

Knee flexion/extension is among the most accurately estimated degrees of freedom for both IMU- and vision-based technologies because of its large sagittal-plane excursion and the relatively constrained hinge-like anatomy of the joint. By contrast, the secondary rotational degrees of freedom are substantially harder to estimate reliably. Their small amplitudes make them vulnerable to cross-talk caused by S2S misalignment in IMU pipelines and to depth ambiguity in monocular vision systems. These secondary rotations are clinically important for the assessment of ligamentous laxity, ACL injury risk, and post-surgical outcomes, yet they remain near the limit of what current wearable and markerless systems can measure robustly in routine applications [51,52,53,54,55,56].

#### 4.2.3. Hip Joint

For the hip, the ISB JCS uses anatomical landmarks such as the anterior superior iliac spines (ASISs) and femoral epicondyles to define pelvic and femoral coordinate systems. Hip motion is described by flexion/extension, abduction/adduction, and internal/external rotation. A key methodological aspect is the estimation of the hip joint center, which is often performed using functional methods.

##### Technology-Specific Challenges

The hip presents distinctive challenges for both technologies. In IMU-based systems, internal/external rotation is particularly sensitive to heading drift in magnetometer-free configurations, and the pelvic segment is difficult to instrument reliably because the anatomical frame must be inferred from a sensor placed on soft tissue rather than directly over bilateral pelvic landmarks [10,53]. In vision-based systems, hip estimation is complicated by frequent occlusion of the pelvis and by the difficulty of inferring the anatomical hip joint center from externally visible landmarks alone. Markerless pipelines that enrich sparse keypoints or incorporate musculoskeletal modeling tend to improve these estimates, especially for pelvis- and hip-related kinematics [15,16].

#### 4.2.4. Spine

In the ISB framework, spinal kinematics concerns the relative motion between adjacent vertebral segments. Motion may be described in terms of flexion/extension, axial rotation, and lateral bending, together with translational components, although practical measurement often requires substantial simplification because of the large number of contributing segments.

##### Technology-Specific Challenges

Spinal kinematics is inherently more complex than single-joint kinematics because gross trunk motion reflects the cumulative contribution of many intervertebral segments. In practice, both IMU- and vision-based systems usually provide a trunk- or lumbar-level approximation rather than true intervertebral motion, which limits interpretation in conditions where segment-specific motion is clinically relevant. Vision-based systems face additional challenges because posterior trunk landmarks are frequently occluded by clothing, chairs, backrests, or the subject’s own upper limbs during functional tasks. IMU-based systems can capture global trunk orientation well, but the validity of segment-by-segment spinal estimates remains more limited and strongly protocol-dependent [10].

#### 4.2.5. Shoulder

The shoulder complex includes the glenohumeral, acromioclavicular, sternoclavicular, and scapulothoracic articulations and provides the largest range of motion of any major human joint. The ISB recommendations define local coordinate systems for the clavicle, scapula, humerus, and thorax using anatomical landmarks on these segments. The principal rotational components include flexion/extension, abduction/adduction, and internal/external rotation, while scapular motions such as upward/downward rotation and anterior/posterior tilting are also considered essential to shoulder function.

##### Technology-Specific Challenges

The shoulder is arguably the most problematic joint complex for both IMU- and vision-based estimation. In IMU systems, the strong soft-tissue artifacts around the scapula and the multi-joint nature of the shoulder girdle make S2S calibration especially challenging. Systematic-review evidence indicates that accuracy varies substantially across joints and computational methods, with scapulothoracic and glenohumeral estimates generally more difficult than simpler hinge-like upper-limb motions [11]. In addition, the ISB-recommended decomposition for some shoulder motions may approach singular configurations, which complicates interpretation near specific postures. For vision-based systems, the scapula is not directly observable from external keypoints, and frequent self-occlusion occurs during overhead or cross-body movements because of overlap between the trunk and upper arm.

#### 4.2.6. Elbow

The humeroulnar joint is typically modeled as a hinge joint, with flexion/extension as the primary degree of freedom, while forearm pronation/supination is associated with the proximal and distal radioulnar joints. Coordinate systems for the forearm are defined using anatomical landmarks such as the ulnar and radial styloids.

##### Technology-Specific Challenges

Elbow flexion/extension is generally estimated with relatively good accuracy by both IMU- and vision-based approaches. Pronation/supination, however, is more problematic. In IMU systems, rotation about the longitudinal axis of the forearm is sensitive to sensor misalignment and soft-tissue motion, whereas in vision-based systems, pronation/supination produces only limited changes in the external contour of the forearm and is therefore difficult to infer reliably from image data alone. Recent markerless musculoskeletal evaluations also suggest that elbow kinematics can be considerably less accurate than lower-limb estimates, particularly when the modeling pipeline depends on sparse or visually ambiguous upper-limb landmarks [11,57].

#### 4.2.7. Hand and Wrist

The ISB JCS for the wrist and hand describes the relative motion among the radius, ulna, carpal structures, and metacarpals. Wrist motion is typically characterized in terms of flexion/extension and radial/ulnar deviation, with neutral postures and anatomically defined axes used as references for angular description.

##### Technology-Specific Challenges

Hand and wrist kinematics remain an emerging frontier for both IMU- and vision-based approaches. The small size of hand segments makes multi-sensor IMU instrumentation cumbersome for fine finger-level analysis, although wrist-worn IMUs can capture gross wrist motion effectively. Vision-based methods have shown greater promise for finger kinematics, but their performance is strongly affected by self-occlusion during grasping and object manipulation. Recent validation studies of video-based finger tracking confirm that accuracy is task-dependent and degrades in configurations with severe overlap or poor visibility of individual digits [58].

## 5. IMU-Based Systems in Motion Analysis

Inertial motion analysis has become a reliable and cost-effective alternative to laboratory optoelectronic motion capture systems for many human-movement applications. Wearable IMU/MIMU systems are portable, relatively inexpensive, and deployable without a dedicated capture volume, enabling assessments in clinics, homes, and real-world environments. These setups offer larger workspaces and longer observation windows than typical optoelectronic systems [5,6,8,59,60,61,62].

At the same time, IMU-based systems are susceptible to several technical challenges: sensor drift, variability in IMU placement across sessions, soft-tissue artifacts (STAs), and magnetic disturbances when magnetometers are used. These factors can corrupt heading estimates and propagate into joint-angle errors [10,60]. These limitations are intrinsic to inertial technology and affect accuracy in both laboratory and field settings.

Specifically regarding drift, defined as the accumulation of small measurement biases that lead to increasing errors when signals are integrated over time, even small orientation errors can translate into large inaccuracies when kinematics are further differentiated or integrated (e.g., to obtain angular velocity, linear velocity, or displacement). This is a critical concern since many clinical questions are driven by changes between sessions rather than single-session absolute agreement with an optical reference [10,60].

For this reason, the accuracy of IMU-based systems should be considered with respect to the entire measurement chain rather than as an intrinsic property of the sensor hardware. This chain includes

- Coordinate definitions and biomechanical models.
- Calibration procedures.
- Sensor fusion and orientation estimation.
- Filtering choices and reporting conventions.

**Figure 1:**
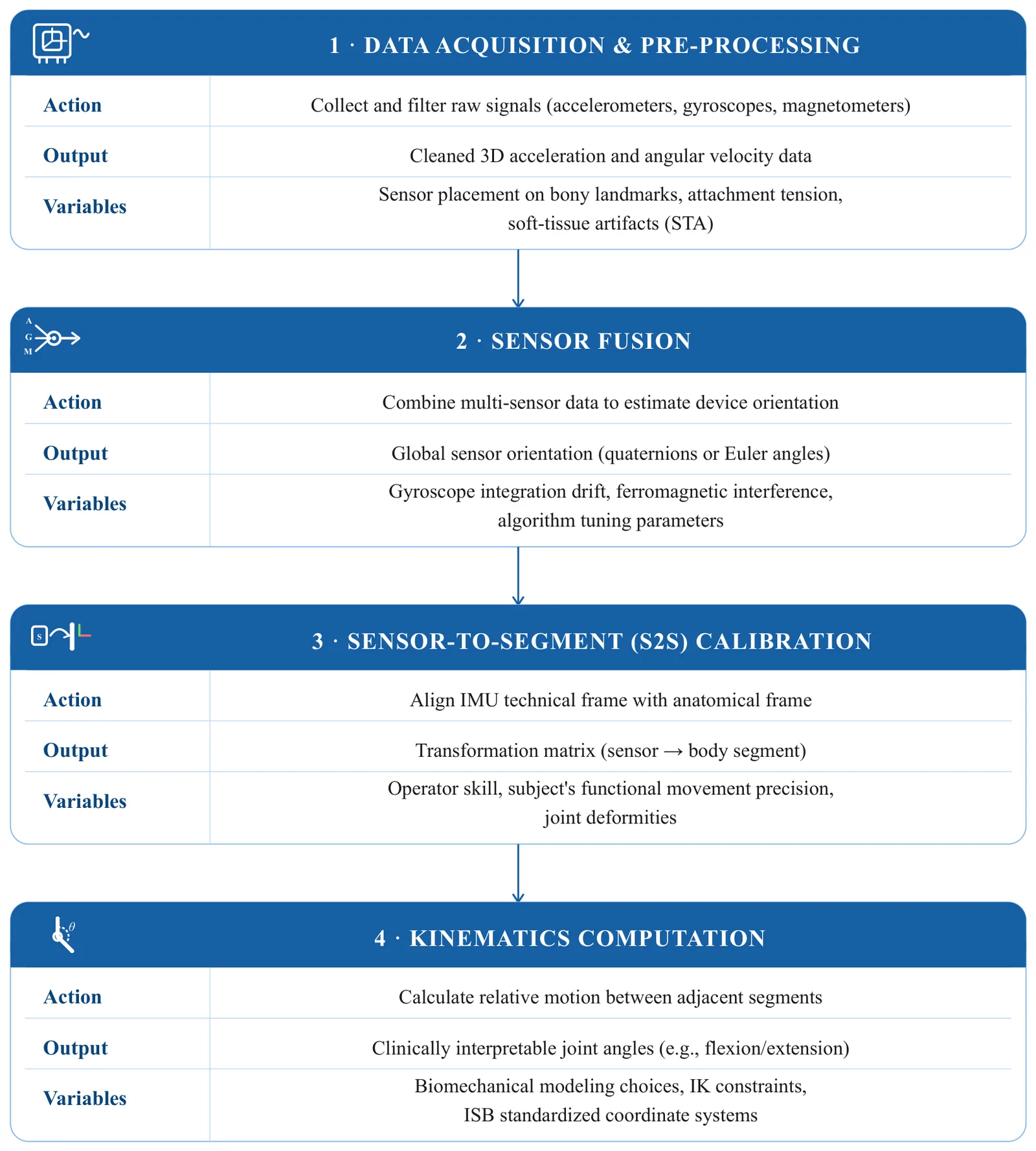
General processing pipeline of IMU-based systems for motion analysis.

All these elements materially influence what is ultimately labeled “accurate” [10]. Recent recommendations from the International Society of Biomechanics (ISB) explicitly emphasize that limited standardization in definitions and reporting can compromise interpretability and reproducibility. This has motivated structured guidance spanning calibration, experimental protocols, model definition, kinematics computation, and quality assessment [9,10]. The overall processing chain is summarized in Figure 1, and the subsections below examine each of its stages in turn.

### 5.1. Sensor Fusion and Orientation Estimation

The first computational step in the IMU pipeline is orientation estimation: converting raw accelerometer, gyroscope, and (optionally) magnetometer signals into the orientation of the sensor in a global reference frame. Gyroscope data provide high-frequency angular velocity that can be integrated to obtain orientation, but this integration accumulates drift over time—the primary technical challenge in IMU-based motion analysis. Accelerometers measure the sum of gravitational and linear acceleration, providing a drift-free estimate of the gravity direction (and thus the inclination, i.e., roll and pitch angles) under quasi-static conditions. Magnetometers provide heading (yaw) information relative to Earth’s magnetic field but are vulnerable to ferromagnetic disturbances from building materials, medical equipment, and electronic devices [10].

Sensor fusion algorithms (SFAs) combine information from these complementary sensors to estimate orientation while mitigating the weaknesses of each individual modality. Following the taxonomy of Nazarahari and Rouhani (2021) [63], who surveyed over 250 publications spanning four decades of research, SFAs can be classified into five families:

1. **Complementary Filters (CFs)** combine low-pass filtered accelerometer/magnetometer data (stable but noisy) with high-pass filtered gyroscope data (precise but drifting) in the frequency domain. The widely adopted Madgwick gradient-descent filter [64] and the Mahony filter [65] belong to this family. CFs are computationally efficient and well-suited for real-time embedded applications.
2. **Extended Kalman Filters (EKFs)** propagate a state estimate (orientation, often parameterized as a quaternion) through a nonlinear process model using gyroscope data, then correct it with accelerometer and magnetometer observations. The EKF remains the most frequently cited SFA in the biomechanics literature [17].
3. **Unscented Kalman Filters (UKFs)** address the EKF’s linearization limitations by using sigma-point sampling, offering improved accuracy for highly dynamic motions at increased computational cost [66].
4. **Complementary Kalman Filters (CKFs)** are hybrid approaches combining the strengths of complementary filtering and Kalman estimation frameworks [67].
5. **Optimization-based methods** solve for orientation by minimizing a cost function that jointly accounts for gyroscope integration and gravity/magnetic field observations, often in a batch or sliding-window fashion [68]

A critical experimental finding is that parameter tuning matters more than algorithm selection. Caruso et al. (2021) [69], in a benchmark of 10 algorithms across three commercial MIMU devices, found orientation errors ranging from $3.8^{\circ }$ to $7.1^{\circ }$ under optimal tuning but noted that errors reported in original algorithm papers (~$1^{\circ }$) were up to ten times lower than those obtained in independent comparative studies. Cereatti et al. (2024) [10] illustrate this powerfully by showing how the Madgwick filter’s single tuning parameter (β) dramatically changes the resulting knee flexion–extension waveform: β=0 (gyroscope-only, maximum drift) vs. β=0.1 (recommended balance) yield visibly different kinematic profiles.

An increasingly important trend is the development of magnetometer-free algorithms. Magnetic disturbances in indoor clinical environments, near medical devices, or during outdoor activities near metallic structures can cause errors exceeding $30^{\circ }$ in the yaw angle. Algorithms that rely exclusively on accelerometer and gyroscope data (6-DOF fusion) avoid this problem entirely. Notable examples include the VQF algorithm [70], which achieves elbow flexion–extension RMSE of approximately $2.2.1^{\circ }$ without magnetometer input. Al Borno et al. (2022) [71] demonstrated that even large magnetic heading errors did not significantly degrade knee flexion angle accuracy when biomechanical model constraints were applied, supporting the feasibility of magnetometer-free clinical pipelines.

### 5.2. Sensor-to-Segment Calibration

Within this chain, one of the most significant and often dominant sources of error is the misalignment between the sensor’s technical coordinate system and the anatomical coordinate system of the underlying body segment. Consequently, there is strong research interest in sensor-to-segment (S2S) calibration, which estimates the transformation between the strapped sensor frame and the segment frame used to compute joint kinematics [9,10]. S2S methodologies can be classified into four main types: manual, static, functional, and anatomical.

In **manual calibration** (manual alignment), the operator positions each sensor so that one or more sensor axes are assumed to coincide with the target anatomical axes (e.g., aligning a sensor edge with the segment’s longitudinal direction). This method is fast, does not require a dedicated calibration movement, and can be used when subjects cannot reliably perform functional tasks [9], but it is operator-dependent and there is a systematic placement bias [9]. In a clinical elbow-angle validation against optical motion capture, manual alignment produced the lowest overall RMSE among three common calibration strategies (manual alignment $\approx 6.3^{\circ }$, functional calibration $\approx 7.2^{\circ }$, and an N-pose approach $\approx 8.2^{\circ }$), leading the authors to recommend manual alignment as a pragmatic default for elbow assessment while noting task/axis dependence and the influence of skin artifacts.

**Static calibration** involves estimating the sensor-to-segment transformation by assuming one or more predefined poses. These methods rely on gravity-based alignment, where accelerometer data are used to align segment axes, for instance, assuming vertical alignment during an upright stance or establishing axes through the comparison of gravity vectors between upright and supine postures [9]. However, subjects may not adopt a perfect neutral pose; then, residual joint angles can be non-zero, and segment axes may not be aligned with the vertical/horizontal directions presumed by the protocol. These kinds of limitations can be amplified due to contractures, deformities, pain-limited postures, or reduced motor control [9]. To reduce calibration errors, clinical frameworks like the Outwalk protocol utilize manual goniometry to account for residual joint flexion during supine IMU alignment. Although this introduces a non-negligible source of uncertainty, inter-rater reliability remains high, with reported RMSE within 1.4–$1.8^{\circ }$.

**Anatomical calibration** reconstructs segment axes using palpable bony landmarks as reference, shifting the main uncertainty toward landmark identification and toward how external landmark geometry is mapped into an anatomically consistent reference frame (often aligned with ISB definitions) [9,10]. Anatomical approaches can be particularly valuable when functional movements are impractical, but they may require trained operators and additional tools, which limits scalability; they may also introduce definitional discrepancies when segment longitudinal axes are inferred from external landmark lines rather than joint-center lines, yielding systematic differences relative to other modeling conventions [9]. Despite these constraints, landmark-based procedures can reach high agreement with optoelectronic references when carefully executed. In an upper-limb protocol based on direct identification of palpable landmarks, validation against camera-based motion capture reported low range of motion (ROM) bias (<$-2.6^{\circ }$), low RMSD (<$4.4^{\circ }$), and very high waveform similarity ($R^{2}>0.995$) for shoulder/elbow angular kinematics while avoiding the need for active calibration movements [72].

**Functional calibration** estimates anatomical axes from task-specific movements, but its accuracy relies heavily on a consistent range of motion and minimal compensations [50]. Lebleu et al. (2020) [51] demonstrated that while optimal calibration achieves low errors relative to optical systems (RMSE $<3.6^{\circ }$) and high reproducibility (ICC >0.91), performance significantly degrades when movements are constrained. Thus, while effective in healthy subjects, it faces scalability challenges in clinical populations with movement deficits.

Given the limitations of single-method calibrations, hybrid procedures combining static and functional elements are increasingly used to improve robustness and feasibility.

### 5.3. Signal Processing, Sensor Placement, and Attachment

The quality of raw IMU signals is strongly influenced by sensor placement on the body and the method of attachment to the skin. Both factors directly affect the magnitude of soft-tissue artifacts (STAs)—the time-varying relative motion between the sensor and the underlying bone—which represent a source of error that cannot be fully eliminated by any algorithmic approach.

**Sensor placement** should follow established guidelines: sensors should be positioned on bony prominences where soft-tissue coverage is minimal, away from joint lines (to avoid large skin deformations during motion), and in areas with low muscle mass [10]. For the lower limb, recommended placements include the flat anterior surface of the tibia (shank), the lateral aspect of the thigh midway between the greater trochanter and lateral epicondyle, and the sacrum or posterior pelvis for the pelvic segment. Uhlenberg and Amft (2024) [73] investigated optimal sensor configurations using a 165-DOF thoracolumbar spine model and found that RMSE for joint angles was smaller at the shoulders compared to the elbows, while shoulder RMSE varied enormously across studies—from $0.2^{\circ }$ to $64.5^{\circ }$—indicating the strong influence of placement and protocol.

**Attachment methods** include double-sided adhesive tape directly on the skin, elastic straps, rigid frames, and integration into garments. A recent literature review recommended that for tasks involving impacts, attachment should be tensioned “as much as tolerable,” areas close to joints and with soft tissues should be avoided, elastic belts should not be used, and low-mass devices are preferred. Quantitatively, bone-mounted vs. skin-mounted accelerometers show peak tibial acceleration differences of approximately 2.1g during running, illustrating the magnitude of STA even under controlled conditions. For less dynamic movements such as walking, ensuring no detectable movement between the sensor and body segment is generally adequate [10].

**Signal pre-processing** typically includes low-pass filtering of raw sensor data (commonly fourth-order Butterworth, cutoff 5–20 Hz depending on the activity), bias estimation and removal for gyroscopes (often performed during a static initialization period of ≥10 s), and detection of invalid data segments due to accidental collisions or loose attachment. The choice of filter parameters must match the frequency content of the movement being analyzed: walking gait contains significant energy up to approximately 6 Hz, while running and jumping require higher cutoffs (15–20 Hz).

### 5.4. Data-Driven Approaches

In recent years, data-driven approaches based on machine learning (ML) and deep learning (DL) have emerged as an alternative paradigm for estimating joint kinematics from IMU data. Instead of following the traditional pipeline (sensor fusion → S2S calibration → geometric joint angle computation), these methods learn a direct mapping from raw or minimally processed sensor signals to joint angles using neural networks trained on large datasets of synchronized IMU and optical motion capture data.

The key architectural families employed in this domain are as follows.

- **Feedforward Neural Networks (FNNs/MLPs)** are the simplest architecture, mapping a window of IMU features to joint angles. Mundt et al. (2021) [74] compared MLPs against recurrent and convolutional models for lower-limb kinematics and reported sagittal-plane correlations above r=0.911, together with a range-normalized rather than absolute error; the absolute value of $4.8^{\circ }$ frequently attributed to this work belongs to the earlier study of the same group [75], and the two should not be conflated.
- **Convolutional Neural Networks (CNNs)** exploit spatial and temporal patterns in multi-channel IMU signals. CNNs were found to be the most frequently used DL architecture for running biomechanics, accounting for the largest share of ML-based approaches in a systematic review by Xiang et al. (2022) [76].
- **Recurrent Neural Networks (RNNs/LSTMs/BiLSTMs)** capture temporal dependencies in sequential IMU data. Long Short-Term Memory (LSTM) networks and their bidirectional variants (BiLSTMs) are particularly effective for gait analysis, where the cyclic nature of movement provides strong temporal structure [77]. TCN-BiLSTM hybrid architectures have achieved improved accuracy over single-architecture baselines [78].
- **Combined DL + optimization frameworks:** Rapp et al. (2021) [79] proposed a system combining deep learning with top-down biomechanical optimization, trained on a large synthetic IMU database derived from clinical motion capture of hundreds of subjects. This approach achieved RMSE $<1.27^{\circ }$ ($\pm 0.38^{\circ }$) for flexion–extension, <$2.52^{\circ }$ ($\pm 0.98^{\circ }$) for abduction–adduction, and <$3.34^{\circ }$ ($\pm 1.02^{\circ }$) for internal–external rotation during walking and running, substantially lower than the errors typically reported for conventional sensor-fusion pipelines.
- **Emerging architectures:** Transformer-based models for sparse inertial pose estimation include physics-informed neural networks (PINNs) that estimate joint angles and moments without explicit ground truth data and cross-modal transfer learning approaches that align IMU embeddings with video representations.

A distinctive advantage of data-driven methods is the possibility of reduced sensor configurations: ML models can estimate multi-joint kinematics from far fewer IMUs than traditional approaches [80,81,82]. Studies have demonstrated accurate joint angle estimation using a single pelvis-mounted IMU for lower-limb angles [80,83], minimal IMU configurations for activity-informed upper-body kinematics using wrist/chest or chest/arm placements [81], or three IMUs with TCN-BiLSTM architectures for hip, knee, and ankle angles [82]. This contrasts with model-based or full-body/sparse IMU approaches requiring approximately 6–17 IMUs for full-body tracking [84,85]. Table 3 summarizes representative data-driven studies. Because these studies do not all share the same target output, the table separates works that estimate continuous joint angles, and are therefore reported in degrees, from works that address movement or fault classification, and are therefore reported as classification accuracy or $F_{1}$ score. These two groups of results are not interchangeable and are not pooled anywhere in this review.

However, generalizability remains the principal limitation: inter-subject models consistently produce higher errors than intra-subject (personalized) models. Transfer learning with small fine-tuning datasets is the most actively investigated solution to this challenge. Additionally, training data quality is fundamental: synthetic IMU data generated from optical motion capture databases dominates but lacks the vibration, impact artifacts, and attachment noise present in real sensor signals.

## 6. Vision-Based Systems in Motion Analysis

Vision-based systems for human pose estimation have become a rapidly evolving alternative to traditional marker-based optical motion capture technologies, especially within the domain of human motion analysis. Unlike traditional systems that require the placement of reflective markers on anatomical landmarks and rely on multiple high-speed infrared cameras, markerless systems aim to estimate the pose of the human body directly from video data without the need for physical markers. These systems utilize computer vision techniques to estimate joint angles in anatomical planes, providing valuable insights into both upper and lower limb movements. The primary advantage of vision-based systems lies in their non-invasive nature, which allows for the capture of human motion without the need for physical markers or sensors attached to the body [88]. This feature is particularly beneficial in clinical settings where patient comfort and ease of use are paramount [88,89]. The pose estimation process in markerless systems may rely on different computational strategies, including generative methods, which optimize the parameters of a 3D body model to match the image data [90]; discriminative methods, which predict joint positions directly from image features using machine learning [91,92]; or hybrid approaches that combine both techniques [93]. Another markerless configuration gaining popularity involves combining a monocular RGB camera with a depth sensor. A notable example is the Microsoft Kinect, a commercial RGB-D camera that allows for real-time 3D body tracking by leveraging both color and depth information [94]. The integration of depth data improves pose estimation, particularly in scenarios with occlusions or complex body configurations [95].

**Figure 2:**
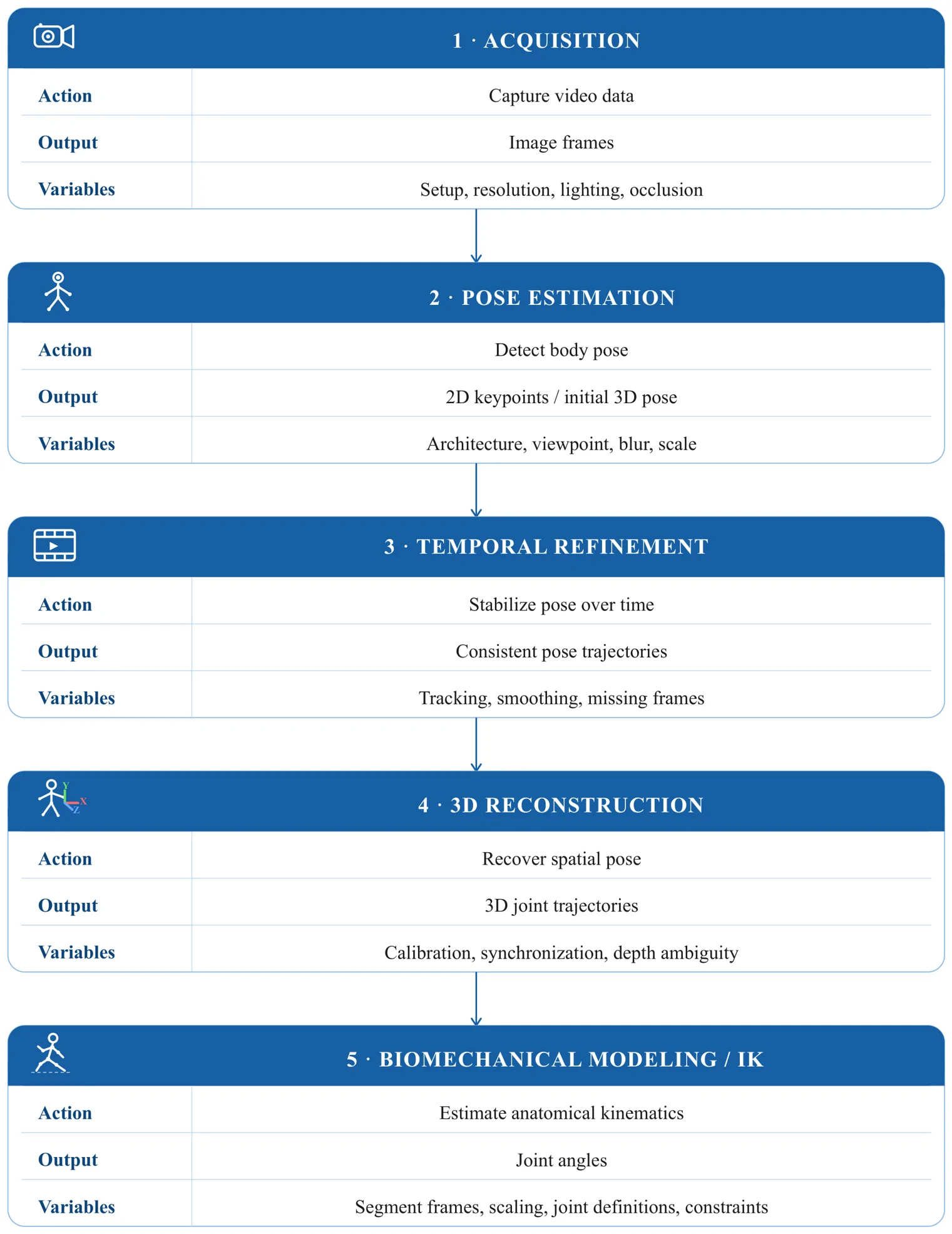
General processing pipeline of vision-based systems for motion analysis.

Furthermore, deploying multiple RGB-D cameras around the capture space can extend the field of view and enhance accuracy through sensor fusion techniques [96]. The corresponding processing chain, from image acquisition to joint-angle estimation, is summarized in Figure 2.

In the simplest configuration, a single RGB camera can also be used to estimate 3D human pose [97,98]. However, this approach faces inherent limitations due to the lack of depth information, which makes the problem under-constrained and more prone to ambiguities [93]. As a result, single-camera systems generally provide lower accuracy compared to multiview setups. Despite their advantages in terms of usability and reduced setup complexity, vision-based systems are not without limitations. One of the primary challenges is motion blur, which can occur during rapid movements and significantly reduce tracking accuracy. Occlusions, caused by overlapping body parts or environmental obstacles, also present major difficulties for reliable pose estimation [90]. Moreover, vision-based systems are generally not wearable, as they require cameras to be placed externally around the capture area to maintain full visibility of the subject’s body throughout the movement [88].

Human pose estimation relies on large annotated datasets, which differ in ways that bear directly on their suitability for biomechanical use. Table 4 provides a comprehensive overview, and the attributes that matter most are discussed below.

A fundamental aspect of human pose estimation datasets is the number of keypoints they annotate. These keypoints represent crucial anatomical landmarks such as the head, torso, and upper and lower limbs. Some datasets, such as COCO [99], provide 17 keypoints, which include major joints like shoulders, elbows, and knees. Others, like Halpe WholeBody [100], extend this to 136 keypoints, incorporating face, hands, and feet annotations for a more detailed representation of human posture. The density and precision of keypoints determine a dataset’s applicability to biomechanics and ergonomics research, where precise tracking of movements is essential for assessing posture, injury risks, and ergonomic efficiency, and it also determines whether distal segments can be resolved at all.

The number of images or frames in a dataset also plays a significant role in its utility. Larger datasets like COCO, with over 200,000 images, allow for robust model training, offering diverse scenarios and backgrounds. In contrast, controlled datasets such as Human3.6M [101] provide around 3.6 million frames but in structured laboratory environments, making them ideal for detailed motion capture but potentially less effective for real-world applications. Datasets like Panoptic [102], with 1.5 million frames, bridge the gap between structured motion capture and real-world diversity by incorporating multi-view annotations and multi-person scenarios.

The number of annotated subjects directly affects diversity and applicability. COCO [99] includes over 250,000 human instances, ensuring a wide variety of body shapes, clothing, and environments. MPII [103], with 40,000 annotated subjects, focuses more on individual action recognition, which is crucial for analyzing specific biomechanical movements. In contrast, datasets such as Human3.6M [101] have only 11 subjects, but their highly detailed 3D annotations make them invaluable for controlled movement analysis.

Another critical factor is the availability of multi-view annotations, and with it the support for 3D rather than merely image-plane estimation. Datasets like Human3.6M [101], 3DPW [104] and Panoptic [102] capture human movement from different angles and provide 3D annotations, which is a prerequisite for models intended to recover joint angles and for applications requiring depth information, such as gait analysis and motion prediction. Single-view datasets such as COCO and MPII, designed primarily for 2D pose estimation, are correspondingly less suitable where depth perception or full-body posture assessment is necessary.

The inclusion of multi-person scenarios is another key differentiating feature. COCO [99] and PoseTrack [105] support multi-person tracking, making them valuable for studying interactions between individuals, such as teamwork dynamics or pedestrian movement analysis. On the other hand, datasets like HumanEva [106] and AIST Dance [107] focus on single-person movements, which are more suited for detailed motion studies in controlled settings, such as rehabilitation and physical therapy applications.

**Table 4:** Comparison of Human Pose Estimation Datasets.

Dataset	Frames/Img	Kpts	Locations	Subjects	Tasks	MV	MP
COCO [99]	200k+	17	Head, torso, limbs	250k+	Multi	No	Yes
MPII [103]	25k+	16	Head, torso, limbs	40k+	Multi	No	No
Human3.6M [101]	3.6M	17	Head, torso, limbs	11	Multi	Yes	No
AIST Dance [107]	3.5k	17	Head, torso, limbs	30	Dance	Yes	No
PoseTrack [105]	10k	17	Head, torso, limbs	4k	Multi	No	Yes
HumanEva [106]	40k+	15	Head, torso, limbs	4	Sport	Yes	No
3DPW [104]	51k	24	Head, torso, limbs	7	Multi	Yes	Yes
Penn Action [108]	158k	13	Head, torso, limbs	2326	Sport	No	No
DensePose [109]	50k+	Dense	Full-body surface	39k+	Multi	No	Yes
FLIC [110]	5k+	10	Head, limbs	5k	Multi	No	No
Halpe [100]	100k+	136	Full body + Face	50k+	Multi	No	Yes
HICO-DET [111]	150k+	17	Head, torso, limbs	47k+	Interact	No	Yes
Panoptic [102]	1.5M	17	Head, torso, limbs	60+	Interact	Yes	Yes

*Note: MV = Multi-View Annotations; MP = Multi-Person Scenario.*

### Models and Architectures for Video-Based Human Pose Estimation

Various models and architectures have been developed to address challenges associated with motion dynamics, occlusions, and variations in capture conditions in video. In practice, video-based human pose estimation is commonly implemented as a multi-stage pipeline rather than a single end-to-end architecture. Typical systems combine

1. Per-frame feature extraction and a prediction head for 2D or 3D estimation.
2. Optional components for temporal consistency (e.g., tracking, smoothing, or sequence modeling).

Depending on the target output, pipelines may also include 2D to 3D lifting, multi-view triangulation, and, in biomechanics-oriented applications, Inverse Kinematics (IK) to estimate joint angles rather than only joint positions.

Within such pipelines, a fundamental component is the feature extraction backbone, which extracts visual representations from each frame. Convolutional backbones (e.g., ResNet [112]) and high-resolution architectures (e.g., HRNet [113]) are frequently used to preserve spatial detail relevant to keypoint localization. HRNet maintains high-resolution representations throughout the network, which supports localization of small joints under viewpoint changes and partial occlusion [113]. Transformer backbones have also been adopted in pose estimation: ViTPose shows that Vision Transformer backbones can provide competitive performance when combined with appropriate pose heads and training procedures [114]. Efficiency-oriented transformer variants (e.g., token clustering in TCFormer ) can reduce computation by prioritizing salient regions, which is relevant for long video sequences where throughput constraints are present [115].

The field of HPE then bifurcates into two primary domains: 2D pose estimation, which predicts joint locations in a two-dimensional plane, and 3D pose estimation, which reconstructs the spatial configuration of joints in three-dimensional space. The transition from 2D to 3D poses significant challenges due to depth ambiguity, occlusions, and varying camera perspectives.

In 2D pose estimation, the objective is to detect keypoints such as elbows, wrists, and knees within an image or video frame. Early methods employed pictorial structures and handcrafted features like Histograms of Oriented Gradients (HOGs). However, these approaches struggled with occlusions and complex poses. The advent of deep learning, particularly Convolutional Neural Networks (CNNs), revolutionized the field by enabling models to learn hierarchical feature representations directly from data. Notable advancements include the introduction of DeepPose, which treated pose estimation as a regression problem using deep neural networks. Subsequent models such as OpenPose [116] and AlphaPose further improved accuracy by refining network architectures and training strategies.

**Figure 3:**
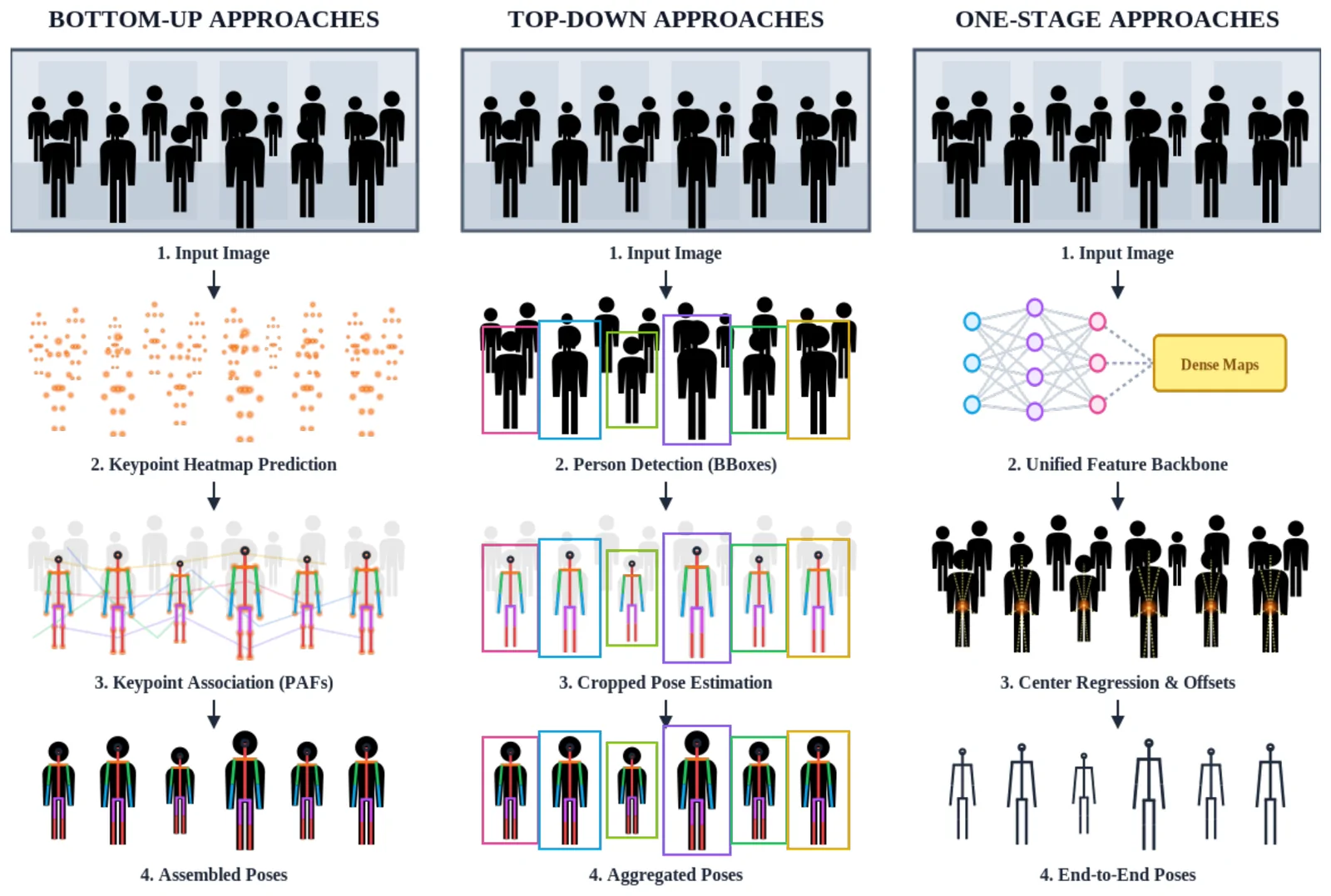
Overview of 2D multi-person pose estimation paradigms. The diagram illustrates the core architectural differences between bottom-up, top-down, and one-stage approaches. Colored boxes denote person instances returned by the detector, and colored segments denote skeletal links. In the bottom-up branch, orange markers are candidate keypoint heatmap responses and the thin curves are the part affinity fields used for grouping; in the one-stage branch, the dashed rays represent center-to-keypoint offset regression. Colors distinguish instances and body segments only and carry no quantitative meaning.

A common categorization of 2D pose estimation methods in multi-person video, illustrated in Figure 3, distinguishes bottom-up, top-down, and one-stage paradigms, which differ in computational scaling and in how person instances are handled.

**Bottom-up** methods first detect keypoints and then group them into person instances; OpenPose associates joints through Part Affinity Fields [116], HigherHRNet addresses scale variation via multi-resolution aggregation [92], and DEKR disentangles keypoint representations to improve localization without a separate person detector [117]. **Top-down** methods first detect persons and then estimate pose within each detection, which generally improves per-person localization in complex poses at a computational cost that grows with the number of individuals; representative families include HRNet [113], the transformer-based ViTPose [114], the RMPE/AlphaPose line [118], and, for real-time deployment, RTMPose [119]. **One-stage** methods such as RTMO predict multi-person keypoints in a single pass, targeting stable throughput in crowded scenes [120].

In practice, the choice among these paradigms is typically determined by constraints such as the number of people, real-time requirements, and the required per-person accuracy.

Temporal processing is often introduced to reduce frame-to-frame variability and to improve continuity under short-term occlusions. One commonly used approach is tracking-based association, in which per-frame detections are linked across time to form identity-consistent trajectories. For example, bottom-up detections produced by OpenPose can be combined with association modules such as PoseFlow to improve continuity without modifying the underlying per-frame estimator [121].

In addition to association, some systems use explicit temporal models. Earlier pipelines frequently used recurrent units (e.g., LSTM/GRU) to represent sequential dependencies, whereas more recent work has used temporal convolutions and transformer-based temporal modeling to support parallel computation and longer temporal contexts. Temporal modeling may be performed either in image space (learning spatiotemporal features from video) or in pose space (operating on sequences of detected keypoints). Pose-space temporal modeling is commonly used when the objective is 3D reconstruction from 2D trajectories.

Although single-video-based methods have matured, they remain constrained by the lack of depth information required for 3D human motion analysis. The field of 3D Human Pose Estimation (3D-HPE) employs diverse problem-solving strategies, primarily categorized by the number of processing stages:

**Single-stage** methods regress 3D pose directly from images, avoiding the error propagation of multi-stage pipelines; examples include LCR-Net++ [122] and UniPose+ [123], which integrate depth regression into the network, multi-view formulations exploiting spatial consistency [124], multi-view pose transformers [125], and TesseTrack for simultaneous reconstruction and person association [126]. They are computationally demanding, require extensive 3D annotation, and are sensitive to lighting and background, since direct image-to-3D regression is a strongly nonlinear problem [14]. **Two-stage** methods instead lift an intermediate 2D representation to 3D, either from pose-aware features such as heatmaps [127] or directly from 2D keypoint coordinates [128,129]. This exploits the maturity of 2D estimation and the abundance of 2D-labeled data but inherits the 2D model’s errors and remains an ill-posed inverse problem, prone to depth ambiguity and degraded by severe occlusion or complex illumination in unconstrained settings.

Another aspect to take into account is the coordinate systems used in 3D-HPE, which can be categorized into two methods: person-centric and camera-centric.

**Person-centric (root-centric)** approaches predict 3D keypoints relative to a central body point, such as the pelvis. This method is primarily used for single-person pose estimation from monocular images, effectively capturing relative joint positions [130]. However, they struggle with multi-person or multi-view scenarios due to the loss of global positional information.
**Camera-centric** methods predict keypoints relative to the camera, enabling the estimation of absolute coordinates. This is crucial for multi-person 3D-HPE, especially with multi-view input, as it accounts for depth and scale variations. Camera-centric coordinates facilitate easier projection across different views, simplifying occlusion handling.

For multi-camera setups, a global or world coordinate system is often preferred. Estimating absolute 3D poses is more advantageous in real-world applications, such as object interaction analysis in retail or augmented reality.

Traditional methods for 3D HPE rely on stereophotogrammetry with a multi-view setup, reconstructing spatial coordinates from synchronized 2D projections via triangulation. Algorithms such as the Direct Linear Transform (DLT) and bundle adjustment are employed to minimize reprojection errors. Multi-view HPE reduces depth ambiguity and can mitigate failures due to occlusion. Multi-view 3D pose estimation can be performed via direct multi-view networks (e.g., VoxelPose [131], TEMPO [132]) or via triangulation-based pipelines that fuse synchronized 2D detections into 3D points. Toolkits such as Anipose implement calibration and triangulation with optional spatiotemporal constraints to produce stable 3D keypoints [133]. Multi-view datasets and capture systems such as Panoptic Studio have been used to evaluate these methods under multi-person interactions and frequent occlusions [134]. Table 5 summarizes the representative models discussed above according to their task, paradigm, input modality, and whether they incorporate temporal modeling, 3D lifting or triangulation, and inverse kinematics.

Pagnon et al. introduced Pose2Sim, a pipeline that integrates 2D keypoints, multi-view triangulation, filtering, and OpenSim Inverse Kinematics (IK) to estimate joint kinematics [16]. Similarly, OpenCap employs a multi-view approach leveraging smartphone video, incorporating temporal learning, specifically LSTM-based marker augmentation, prior to OpenSim IK processing [15,142]. OpenSim itself remains a critical tool for musculoskeletal modeling and IK and is widely utilized downstream of video-based 3D reconstruction [142].

Crucially, the definition and density of predicted landmarks significantly influence biomechanical accuracy. Methods such as SynthPose [141] (within the OpenCapBench framework) address this by adapting pose models to generate denser or biomechanically aligned keypoint sets. These optimized sets enhance downstream kinematics when combined with triangulation and IK workflows.

Overall, current video-based pose estimation systems can be described as modular combinations of 2D estimation (top-down, bottom-up, or one-stage), temporal consistency components, 3D reconstruction (lifting or multi-view fusion), and, when required, IK-based biomechanical modeling, selected according to the intended measurement output (2D tracking, 3D joint trajectories, or joint angles and dynamics).

## 7. Evaluation Metrics

In human motion analysis, it is crucial to clarify that the evaluation metrics described herein are used to assess the validity, accuracy, and reliability of the measurement systems compared to a ground truth, rather than representing synthetic clinical indices of patient kinematics. Whether the system under investigation is based on wearable inertial sensors (IMUs) or markerless computer vision techniques, the primary objective of this validation is to assess the agreement of the estimated kinematic outputs (specifically, joint angles) against a reference gold standard (e.g., optoelectronic marker-based systems). Consequently, this framework focuses exclusively on standard biomechanical validation metrics applied to both IMU- and vision-based methodologies.

To quantify system performance, the errors are computed by comparing the estimated joint angles (${\hat{\theta }}_{i}$) against the reference joint angles ($\theta_{i}$). The metrics employed evaluate either the absolute accuracy of the system or its reliability and agreement, as detailed in Table 6. Instead of focusing on absolute joint positions in space, biomechanical evaluation emphasizes kinematic consistency and inter-trial reproducibility. Metrics such as the Root Mean Square Error (RMSE) quantify the global error by measuring the quadratic mean difference between the estimated joint angles and those obtained from a gold standard system. Similarly, the Mean Absolute Error (MAE) provides an alternative measure of accuracy by computing the absolute mean difference, offering a more robust evaluation that does not excessively penalize outliers.

Beyond accuracy, assessing the repeatability and consistency of measurements is crucial. The Intraclass Correlation Coefficient (ICC) is widely used to determine how consistent joint angle estimations are when repeated across trials, subjects, or operators. A value close to 1 indicates high reproducibility, ensuring that measurements are stable over time. Additionally, the Coefficient of Variation (CV) quantifies the relative variability of the joint angle measurements with respect to the mean value, facilitating comparisons across different subjects or measurement techniques. To evaluate agreement between different measurement systems, statistical approaches such as the Bland–Altman Plot are commonly applied. This method visually represents the distribution of errors by plotting the differences between two measurement methods against their mean values, revealing potential systematic biases or random discrepancies. Furthermore, the Pearson’s Correlation Coefficient (r) assesses how well the estimated joint angles follow the trends of the gold standard, ensuring that the system accurately captures dynamic changes in movement patterns. Finally, mean difference (MD) is used to determine systematic bias by averaging the differences between the estimated and reference joint angles. This metric is particularly useful for identifying consistent over- or underestimations in joint angle calculations, which could indicate calibration errors or methodological inconsistencies.

To provide a broader context on the specific focus of this study, it must be noted that purely computer-vision-oriented spatial metrics (such as the Percentage of Correct Keypoints or Mean Per Joint Position Error) fall outside the scope of this kinematic evaluation and are therefore not utilized. Indeed, evaluation metrics differ significantly depending on whether the system is based on computer vision techniques or biomechanical motion capture. While both approaches aim to quantify human movement, they rely on distinct methodologies and focus on different aspects of motion representation.

Computer-vision-based human pose estimation primarily evaluates the accuracy of joint position reconstruction in 2D or 3D space. Metrics in this category, such as the Percentage of Correct Parts (PCP), determine the accuracy of limb detection by considering a limb correctly estimated if the distance between predicted and true joint positions is within half the limb’s length. However, PCP tends to penalize shorter limbs more severely. To address this, the Percentage of Correct Keypoints (PCK) evaluates the accuracy of keypoint localization within a specified threshold, often set relative to the head bone length (PCKh@0.5) or torso diameter (PCK@0.2), providing a more balanced assessment across different limb lengths. The Percentage of Detected Joints (PDJ) further refines this by measuring the detection rate of joints within a certain fraction of the torso diameter, alleviating biases against shorter limbs. For 3D pose estimation, the Mean Per Joint Position Error (MPJPE) calculates the average Euclidean distance between predicted and ground truth joint positions, offering a direct measure of positional accuracy. Additionally, the Object Keypoint Similarity (OKS) metric, utilized in benchmarks like COCO, assesses keypoint prediction accuracy by considering the scale of the person and the distance between predicted and true keypoints, normalizing the precision across different body parts. Recent advancements in 3D human pose estimation have introduced novel metrics and methodologies to enhance accuracy and computational efficiency. The Pose SIMilarity (PSIM) metric leverages perceptual mechanisms by incorporating structural, positional, and intrinsic similarities to capture complex spatial and temporal dependencies in human postures. Additionally, the Triangulation Residual loss (TR loss) enforces global multiview geometric consistency for self-supervised learning, surpassing traditional methods that rely on pairwise consistency and costly epipolar constraints.

These computer vision spatial metrics are summarized in Table 7 for completeness.

## 8. Measurement Errors and Accuracy

Accuracy in assessing joint kinematics is crucial because small differences can alter clinical interpretation of the subject (e.g., range of motion deficits, asymmetries, and coordination patterns). Consequently, most validation studies compare IMU or vision-based joint angles against a reference system, most commonly marker-based optical motion capture (OMC) in controlled laboratory environments. Although OMC is widely treated as a laboratory reference, it is not error-free: instrumental errors (camera calibration, reconstruction), data-processing choices (filtering, gap filling), and marker-related issues propagate into kinematic estimates and can affect repeatability and agreement analyses [2].

Reported accuracy varies across joints, planes, and tasks, but that variability should not always be read as meaning that lower accuracy makes a method unsuitable for a given purpose. The most widely used clinical reference comes from the systematic review of McGinley et al. [143], which treats sagittal-plane errors up to about $2^{\circ }$ as acceptable, 2–$5^{\circ }$ as tolerable but to be weighed during interpretation, and errors above $5^{\circ }$ as potentially misleading.

To clarify the underlying purpose and provide a more nuanced interpretation of acceptable measurement errors, we present a range of different clinical situations. This cleanly separates use cases into two distinct classes. When critical clinical decisions must be taken, such as rehabilitation after ACL reconstruction surgery [144], injury-risk screening [145], or surgical planning in cerebral palsy, where specific thresholds apply [146], a strict $\pm 5^{\circ }$ error margin is essential. This requires controlled inertial or optoelectronic systems, though carefully calibrated wearables can also meet this standard for specific variables [147]. Conversely, applications such as tremor severity scoring [148,149,150], fall-risk screening [151,152], gait-event detection [153] and routine range-of-motion follow-ups benchmarked against goniometry [154,155,156] are driven by accessibility rather than laboratory-level accuracy. Table 8 illustrates this range of situations in more detail, linking each application to its required output, acceptable error tolerance and most suitable technology.

IMU-derived joint angles are typically obtained by (1) estimating each sensor’s orientation via sensor fusion, (2) mapping sensor frames to anatomical segment frames (sensor-to-segment alignment), and (3) computing joint angles via a kinematic model (often with Inverse Kinematics constraints). Errors can originate at each step.

Gyroscope integration drift accumulates over time. When magnetometers are excluded (magnetometer-free pipelines), heading drift can dominate transverse-plane errors, particularly for internal/external rotation. In treadmill walking, elevated hip internal/external rotation error was explicitly linked to heading drift in a magnetometer-free approach [53]. In contrast, methods designed for long recordings may explicitly quantify and mitigate drift: OpenSense reported near-zero drift slopes (approximately −0.14 to 0.17 deg/min) while maintaining moderate RMS differences vs. OMC over long durations [71].

Indeed, misalignment between sensor and anatomical frames produces systematic offsets and ROM distortions. Studies comparing functional calibration procedures show that the choice and quality of calibration motions affect accuracy, particularly for distal segments where small axis errors are amplified at the joint [51]. Beyond calibration strategy, the physical placement of IMUs also influences bias and RMSE; a dedicated sensor placement optimization study demonstrated that errors depend on sensor location and the movement sub-task being analyzed, highlighting a practical source of variability in clinical and field deployments [160].

Moreover, skin-mounted sensors move relative to the underlying bone, especially during dynamic tasks. This induces phase-dependent and task-dependent error components (e.g., different error behavior in stance vs. swing). Such effects are often implicated when joint-angle trajectories show systematic over- or underestimation during specific gait phases [53]. While this challenge is widely recognized, the degree of its impact varies by joint, attachment method, and task dynamics.

Joint models, constraints used in Inverse Kinematics, and limited excitation of certain DOFs can create observability limitations. This is particularly relevant in gait, where some DOFs (e.g., transverse-plane rotations) may be weakly excited and thus difficult to estimate robustly from IMU data alone, increasing sensitivity to drift, calibration errors, and modeling assumptions [53]. Across lower-limb gait validations, IMU errors are commonly reported in the few-degree range under controlled conditions with careful calibration, but they often increase for transverse-plane rotations, distal joints, or less controlled setups.

Inertial Measurement Unit (IMU)-based approaches for human joint kinematics have evolved through successive methodological refinements in calibration, sensor fusion, and biomechanical modeling. The following representative studies illustrate how experimental design and processing assumptions influence accuracy, reproducibility, and drift stability. Lebleu et al. (2020) [51] investigated how different sensor-to-segment calibration procedures affect the estimation of lower-limb joint angles during locomotion. Using seven x-IMU units and comparing four calibration movements against an eight-camera optoelectronic reference, they reported a maximum sagittal RMSE of $3.6^{\circ }$ during level walking, rising to $12.8^{\circ }$ on stair descent and amplitude differences under $3.4^{\circ }$. The study also found excellent repeatability (ICC >0.85; SEM $<2.2^{\circ }$), demonstrating that calibration protocol selection is a critical determinant of both absolute accuracy and intra-session reliability [51]. AlBorno et al. (2022) [71] developed OpenSense, an open-source framework for long-duration lower-limb kinematic tracking based on IMU data and Inverse Kinematics. In comparison with optical motion capture, they observed median RMS differences between $3^{\circ }$ and $6^{\circ }$ for most joints and quantified drift by estimating the error-growth slope. Their reported drift rates, approximately −0.14 to 0.17 deg/min, confirmed high long-term stability, an essential feature for extended or field-based monitoring [71]. In a related validation, Potter et al. (2022) [161] evaluated an Error-State Kalman Filter (ESKF) approach across multiple gait conditions. Using seven IMUs, they found RMS differences generally below $5^{\circ }$ for sagittal- and frontal-plane degrees of freedom and spatiotemporal errors under 0.13 m for stride length and step width. This work highlighted that fusion algorithms integrating kinematic constraints can generalize well across walking styles when appropriately tuned [161]. McGrath and Stirling (2022) [53] focused on a magnetometer-free IMU configuration for treadmill gait to avoid magnetic interference. While this design prevented heading disturbances, it revealed the characteristic trade-off with drift: bias-removed RMSE was $3.77^{\circ }$ (knee flex/ext), $3.70^{\circ }$ (hip flex/ext), $4.56^{\circ }$ (hip ab/adduction) and $6.27^{\circ }$ (hip rotation), with the corresponding absolute values being $7.87^{\circ }$, $8.48^{\circ }$, $14.33^{\circ }$ and $14.39^{\circ }$. The authors linked the larger rotational errors directly to accumulated yaw drift and the non-hinge nature of hip mechanics [53].

Earlier, Palermo et al. (2014) [50] assessed the accuracy and repeatability of a body-to-sensor calibration for inertial gait analysis. They achieved joint-angle accuracy and repeatability below $4^{\circ }$ for most DOFs but noted substantially higher variability for ankle internal–external rotation (≈$9.7^{\circ }$ accuracy; $7.2^{\circ }$ repeatability). This confirmed the persistent difficulty of distal transverse-plane estimation, where soft-tissue artifacts and small rotation amplitudes amplify alignment errors [50].

Building on these foundations, Carcreff et al. (2022) [162] proposed a minimal functional calibration method using seven IMUs for full 3D lower-limb kinematics. Across three calibration tasks and at least eight walking trials per participant, they reported a grand mean RMSE of $7.5^{\circ }$ pooled over eleven kinematic variables, a centered RMSE of $4.0^{\circ }$, and correlation coefficients above 0.85 for sagittal-plane motion, but larger amplitude deviations (~$7^{\circ }$). Their findings illustrate the practical compromise between simplified calibration procedures and the resulting ROM bias [162].

Cerfoglio et al. (2023) [163] evaluated a medical-grade IMU system against an optoelectronic reference during a set of prescribed rehabilitation exercises of the trunk and lower limbs. Their validation focused primarily on clinically interpretable outcomes (especially ROM), reporting RMSE on ROM from $3.68^{\circ }$ (hip flexion) to $14.55^{\circ }$ (trunk anterior flexion). Importantly, they emphasized that performance was task-dependent and that systematic discrepancies could arise from sensor misplacement and model assumptions, reinforcing the idea that clinical robustness depends as much on standardized placement protocols as on the estimation algorithm itself [163].

Beyond individual validation papers, the evidence base for IMU joint-angle accuracy has also been synthesized at the review level. Fang et al. (2023) [11] highlighted that the upper limb, especially the shoulder girdle, remains substantially more difficult than lower-limb gait. Across the reviewed studies, humerothoracic DOFs could achieve differences around ~$4^{\circ }$ under favorable conditions; elbow flexion/extension errors could be as low as ~$2^{\circ }$, whereas glenohumeral motion often remained ≥$6^{\circ }$ and scapulothoracic motion frequently exceeded >$10^{\circ }$. These patterns were interpreted as a consequence of (i) strong soft-tissue artifacts around the shoulder, (ii) the complexity of scapular biomechanics, and (iii) the sensitivity of computed angles to anatomical-frame definitions [11]. Finally, comparability across IMU studies is strongly influenced by how calibration, kinematic definitions, and quality assessment are reported. Recent ISB recommendations explicitly emphasize consistent reporting for definitions, estimation procedures, protocols, and quality assessment to improve reproducibility and cross-study comparability in wearable inertial measurement applications [10].

More recently, IMU-based human motion analysis has also been extended through machine learning and deep learning approaches. These methods aim either to estimate joint kinematics directly from inertial signals or to infer clinically or sport-relevant movement outcomes without fully explicit biomechanical reconstruction. Mundt et al. (2021) [74] compared three neural network architectures, multi-layer perceptron (MLP), LSTM, and CNN, for estimating lower-limb joint angles and moments from simulated IMUs and showed that the simpler MLP achieved performance comparable to the more complex temporal models; that study reports range-normalized rather than absolute error, with the frequently quoted $4.8^{\circ }$ belonging to the earlier work of the same group [75]. Rapp et al. (2021) [79] proposed a combined deep learning and optimization framework trained on a large synthetic dataset and reported state-of-the-art lower-limb flexion/extension estimation with RMSE below $1.27^{\circ }$ using seven simulated IMUs, with the inertial signals being derived from the same marker trajectories used as ground truth [79]. Other studies have focused less on continuous angle reconstruction and more on event or movement classification. For example, Anand et al. (2017) [86] showed that a hybrid BLSTM+CNN architecture using a single wrist-mounted sensor could classify swing-sport actions with 93.6–93.8% accuracy [86], while Taborri et al. (2019) [87] evaluated machine learning classifiers for detecting faults in race walking from seven IMUs, highlighting the strong performance of Random Forest and SVM-based approaches for identifying loss-of-contact and knee-bent events [153]. In parallel, recent hybrid temporal architectures combining Temporal Convolutional Networks (TCNs) and BiLSTMs have aimed to reduce the sensor count to as few as three IMUs while maintaining high sagittal-plane accuracy, with measured-signal implementations reporting mean RMSE of $9.47^{\circ }$ (hip), $8.41^{\circ }$ (knee) and $5.21^{\circ }$ (ankle) [82]. Together, these studies suggest that data-driven IMU pipelines can complement conventional calibration- and model-based workflows, although their generalizability remains strongly dependent on the representativeness of the training data, the sensor configuration, and the target biomechanical variable.

A practical point for interpretation is that studies differ substantially in whether they evaluate 2D vs. 3D kinematics. A 2D setup can be simpler and still provide clinically relevant outcomes (e.g., spatiotemporal parameters, sagittal-plane angles), whereas 3D analysis typically requires multi-view capture and depends strongly on camera calibration, reconstruction geometry, and consistent visibility across views [2]. Vision-based joint kinematics typically then involve (1) 2D keypoint detection, (2) optional multi-view triangulation or model-based 3D lifting, and (3) mapping to a biomechanical representation to compute angles. The following error sources recur across the included markerless literature.

**(a) Camera calibration, reconstruction geometry, and processing.** Instrumental/camera errors propagate through calibration and reconstruction pipelines. The stereophotogrammetry error literature highlights how calibration and processing (including filtering and handling missing data) affect kinematic quantities and their reliability [2]. These issues remain relevant for markerless systems because 3D reconstruction accuracy depends strongly on camera geometry, resolution, and consistent synchronization.

**(b) Occlusion and keypoint mis-detection.** Occlusion is among the strongest practical limitations. In fine-motor tracking such as finger kinematics, reduced agreement has been linked directly to occlusion during specific tasks (e.g., radial walking and MCP flexion), where self-occlusion and object interactions compromise keypoint visibility and reliability [58].

**(c) Video configuration: viewpoint, distance, and resolution.** Multi-view systems can reduce depth ambiguity, but performance depends on camera layout and resolution. A stereoscopic OpenPose-based approach reported trajectory error sensitivity to camera distance, resolution, and gait direction, with best-case errors around ~20 mm and worst-case around ~60 mm under different configurations [164]. This reinforces that “system accuracy” is often a property of the entire capture geometry, not only the pose estimation network.

**(d) Movement amplitude, motion blur, and temporal dynamics.** Markerless performance can be movement-dependent. A study evaluating spontaneous body kinematics estimation from video reported that accuracy was weaker for low-amplitude movements and stronger for larger movements (e.g., >∼10 cm), indicating that small-amplitude motions are more likely to be lost in detector noise, quantization, or ambiguity in keypoint placement [165]. This is particularly relevant when clinical interpretation depends on subtle joint excursions.

**(e) Model assumptions and system comparability.** Even when keypoint detection is stable, differences in biomechanical models (segment definitions, joint centers, constraints) can drive systematic discrepancies. In high-dynamic sport tasks such as the snatch, discrepancies between markerless and marker-based systems were attributed not only to measurement issues but also to differing modeling assumptions and processing pipelines (e.g., joint center definitions, constraints) [166]. Similarly, integrating markerless trajectories with musculoskeletal models introduces additional sources of error from scaling, model fitting, and Inverse Kinematics constraints [57]. Vision-based (markerless) systems report a broad range of accuracy because performance is jointly determined by task complexity, capture geometry (single-view vs. multi-view, calibration, resolution), and whether the target is 2D sagittal-plane outputs or full 3D joint angles. In general, the literature shows that markerless methods can reach clinically useful precision for certain outcomes (especially spatiotemporal parameters and sagittal-plane gait angles in controlled views), but errors grow rapidly when occlusion, depth ambiguity, and high dynamics are introduced.

A clear example of the “best-case” scenario is provided by Stenum et al. (2021) [52], who evaluated a single-camera OpenPose workflow for overground gait analysis against simultaneous 3D motion capture. They reported MAE ≈0.02 s for temporal parameters and 0.049 m for step length at the step level, with improved accuracy when averaging per subject (0.01 s and 0.018 m). For sagittal joint angles, they found hip MAE $4.0^{\circ }$, knee MAE $5.6^{\circ }$, and ankle MAE $7.4^{\circ }$ [52]. When moving from 2D to 3D, error becomes much more dependent on the camera configuration and the conditioning of reconstruction. Zago et al. (2020) [164] demonstrated this configuration sensitivity in a stereo-vision setting, reporting joint-*position* trajectory errors on the order of ~20–60 mm, step length underestimation of ~1.5 cm, and stance/swing timing errors of approximately ~0.02 s/0.05 s under favorable setups, with noticeable variation across configurations [164]. Clinical environments introduce additional constraints, variable lighting, assistive devices, atypical gait patterns, and markerless validation, which often shift from joint angles to more robust spatiotemporal endpoints. Lonini et al. (2022) [156] applied DeepLabCut to videos of stroke survivors and validated derived gait parameters against GAITRite^®^. They reported timing errors within 0.04±0.11 s, cadence mean error −0.25±3.9 steps/min, and walking speed mean error −0.02±0.11 m/s, with better agreement for stance/double-support than swing time [156]. The influence of occlusion becomes even more dominant for distal segments and fine-motor tasks. Gionfrida et al. (2022) validated monocular OpenPose for finger joint angles against marker-based motion capture, reporting overall errors below $11^{\circ }$, but with task-dependent mean differences rising to $5.03^{\circ }$ (radial walking) and $6.82^{\circ }$ (MCP flexion) in conditions where occlusion was more severe [58]. High-dynamic sport actions amplify all of these vulnerabilities. Thiele et al. (2024) compared markerless video-based systems (Theia3D/Contemplas) against Vicon during a weightlifting snatch and reported joint-center differences on the order of centimeters (~4.7±1.2 cm lower limb; ~5.7±1.5 cm upper limb) alongside lower-limb joint-angle RMSD around ~11–$14^{\circ }$ depending on the plane [166]. Finally, when markerless trajectories are passed through musculoskeletal modeling, errors become strongly joint-dependent. Auer et al. (2024) compared video-based motion capture (VMC) to marker-based motion capture (MMC) within a musculoskeletal modeling workflow and reported MAE values around $4.8^{\circ }$ (shoulder), $6.8^{\circ }$ (hip), and $3.5^{\circ }$ (knee), but substantially larger errors for hand ($15.0^{\circ }$) and especially forearm pronation/supination ($37.4^{\circ }$; the figures 13.7 and 27.7 are that study’s error normalized to range of motion, not degrees) [57]. Recent markerless studies also suggest that biomechanically informed reconstruction pipelines may improve agreement with reference systems. In particular, Pemasiri et al. [167] proposed a markerless gait-analysis workflow designed to recover biomechanically meaningful variables from 3D human reconstruction, reporting strong agreement for key gait parameters and supporting the idea that anatomically structured pipelines may reduce some of the ambiguity of pose-only approaches. Likewise, Li et al. [168] showed that multi-camera markerless capture can provide useful kinematic information even in sport-specific tasks, although the validity of fast movements remains sensitive to frame rate, occlusion, and the dynamic complexity of the action. These studies reinforce the idea that the performance of markerless systems depends not only on the pose-estimation backbone, but also on reconstruction strategy, camera synchronization= and task-specific motion characteristics.

Across the literature as a whole, IMUs often remain robust under visual challenges (occlusion, clutter, variable lighting) but require careful calibration and drift management, while vision systems are non-contact and fast to deploy but are more sensitive to camera setup, occlusion, and movement characteristics [53,164,165,169].

A consolidated comparison of the individual validation studies is provided in Table 9, Table 10, Table 11 and Table 12, one per outcome class. Because these quantities do not share a measurand—an angular error in degrees, a positional error in millimeters, and a timing error in seconds are not commensurable—they are reported in separate tables and are never compared across them.

### Studies Comparing Both Modalities in the Same Participants

All of the evidence discussed so far is indirect: it compares values obtained in different laboratories, on different participants, with different protocols and references. A small number of studies address this directly by instrumenting the same participants with both technologies during the same trials, referenced to a common standard. These are considerably more informative for technology selection, and they are summarized in Table 13.

The most directly interpretable study is that of Ma et al. [176], in which seven participants walked overground while inertial sensors, optical markers and OpenCap video recorded the same trials, with both candidate systems referenced to the marker-based standard through the same OpenSim inverse-kinematics model. The central result is that neither modality dominates: the inertial system was more accurate at the knee ($5.74\pm 0.80^{\circ }$ vs. $7.36\pm 3.14^{\circ }$) and marginally at the ankle ($7.47\pm 3.91^{\circ }$ vs. $8.20\pm 3.00^{\circ }$), whereas the markerless system was more accurate for pelvic tilt ($4.28\pm 1.47^{\circ }$ vs. $5.49\pm 2.22^{\circ }$), hip adduction ($4.06\pm 0.78^{\circ }$ vs. $6.10\pm 1.35^{\circ }$) and hip rotation ($4.82\pm 2.30^{\circ }$ vs. $6.09\pm 1.74^{\circ }$). Hip flexion and pelvic rotation did not differ significantly, and for pelvic obliquity, the inertial system was numerically better without reaching significance. One qualification applies when these values are read alongside the others in Table 13: Ma et al. computed RMSE on ensemble-averaged waveforms, which yields systematically smaller values than the per-stride or per-trial RMSE reported by Bertozzi et al. and Edwards et al.

Edwards et al. [177] evaluated markerless, inertial and depth-camera systems against a common optoelectronic reference in thirty adults performing jumps, squats, lunges and cutting manoeuvres, and reported lower RMSE for the markerless system than for the inertial system in every plane, with the transverse plane exhibiting extreme upper bounds for both ($56.6^{\circ }$ and $116.8^{\circ }$ respectively). In the opposite direction, Bertozzi et al. [178] evaluated the same two technologies during a cognitively cued landing task and found the inertial system substantially closer in the sagittal plane, particularly at the hip (13.8–$15.6^{\circ }$ vs. 21.6–$23.7^{\circ }$), with the two modalities comparable in the frontal and transverse planes. In contrast, for neck and trunk kinematics during a simulated surgical task, Zhang et al. [179] found the inertial system to be clearly superior (2.3–$3.9^{\circ }$ vs. 5.5–$8.7^{\circ }$). Ceriola et al. [169] evaluated inertial sensing, a blob-analysis approach and four DNN-based markerless methods (AlphaPose, TCFormer, RTMPose, MediaPipe) in the same participants against a Vicon reference and likewise favored inertial sensing: no vision-based method allowed reliable ankle measurement in that configuration, while RTMPose and MediaPipe came closest at the hip and knee, with minimum range-of-motion errors ($\varepsilon_{\mathrm{ROM}}$) of $3.1\pm 1.8^{\circ }$ and $3.5\pm 1.9^{\circ }$ at the hip and $4.0\pm 3.7^{\circ }$ and $4.8\pm 4.3^{\circ }$ at the knee. A related but weaker design is that of D’Antonio et al. [172], who compared a two-webcam OpenPose workflow against inertial sensors rather than against a laboratory standard, reporting sagittal joint-angle errors from $1.6\pm 0.3^{\circ }$ under optimal visibility to $14.0\pm 1.8^{\circ }$ when visibility degraded; since neither system there is a reference, those figures express disagreement between two candidate technologies rather than error. For spatiotemporal parameters, Nassajpour et al. [180] found a foot-mounted inertial sensor and a depth camera to be broadly comparable against an instrumented walkway in older adults.

Taken together, these studies support a conclusion that indirect comparison cannot establish: under matched conditions, the two modalities are not separated by a uniform accuracy gap, and which one is preferable depends on the specific degree of freedom, the segment involved, and the dynamics of the task. More studies of this design, with larger samples and a wider range of tasks, would be the single most useful addition to this literature. Fusing IMU- and vision-based methods has improved accuracy in individual studies: a quaternion-based visual–inertial fusion recovered knee flexion to $3.96^{\circ }$, against $5.04^{\circ }$ for the inertial sensor and $14.76^{\circ }$ for the depth camera alone [181]. Systematically evaluating fusion-based methods is, however, beyond the reach of the present review and is one of its limitations: the outcomes these studies report are heterogeneous [182,183,184,185,186,187,188], and datasets in which both modalities are captured on the same trials remain scarce. Synchronized multimodal datasets of the kind now beginning to appear [189] are the prerequisite for exploring this direction further.

**Table 13:** Studies in which IMU- and vision-based systems were evaluated in the same participants performing the same tasks against a common reference, reported per joint and plane. A lower value indicates closer agreement with the reference; all values are given to one decimal place.

Study	*n*	Task/Reference	Joint	Plane/DOF	Metric	IMU	Vision
Ma (2025) [176]	7	Overground walking; marker-based via OpenSim IK	Pelvis	Sagittal (tilt)	RMSE	5.5 ± 2.2°	4.3 ± 1.5°
			Pelvis	Frontal (list)	RMSE	3.9 ± 1.5°	5.3 ± 1.0°
			Pelvis	Transverse	RMSE	2.9 ± 1.8°	2.8 ± 1.4°
			Hip	Sagittal	RMSE	5.2 ± 1.3°	6.2 ± 2.8°
			Hip	Frontal	RMSE	6.1 ± 1.4°	4.1 ± 0.8°
			Hip	Transverse	RMSE	6.1 ± 1.7°	4.8 ± 2.3°
			Knee	Sagittal	RMSE	5.7 ± 0.8°	7.4 ± 3.1°
			Ankle	Sagittal	RMSE	7.5 ± 3.9°	8.2 ± 3.0°
Bertozzi (2025) [178]	30	Jump–land–jump; optoelectronic	Hip	Sagittal	RMSE	13.8–15.6°	21.6–23.7°
			Hip	Frontal	RMSE	4.9–6.0°	5.0–5.4°
			Hip	Transverse	RMSE	8.5–11.6°	8.4–9.5°
			Knee	Sagittal	RMSE	6.1–6.5°	6.9–7.8°
			Knee	Frontal	RMSE	5.7–6.8°	—
			Ankle	Sagittal	RMSE	7.2–11.5°	9.9–11.1°
Edwards (2025) [177]	30	Jump, squat, lunge, cut; optoelectronic	Lower limb	Sagittal	RMSE	8.1–28.4°	3.2–15.7°
			Lower limb	Frontal	RMSE	3.3–17.0°	2.1–9.1°
			Lower limb	Transverse	RMSE	5.1–116.8°	3.2–56.6°
Zhang (2022) [179]	10	Simulated surgery; optoelectronic	Trunk	Sagittal	RMSE	2.3 ± 1.1°	5.5 ± 2.1°
			Trunk	Frontal	RMSE	2.5 ± 1.2°	5.6 ± 3.3°
			Trunk	Transverse	RMSE	3.6 ± 1.8°	8.7 ± 4.1°
			Neck	Sagittal	RMSE	3.6 ± 2.2°	6.1 ± 3.2°
			Neck	Frontal	RMSE	3.9 ± 2.0°	7.0 ± 4.2°
			Neck	Transverse	RMSE	3.6 ± 2.1°	7.3 ± 2.7°
Ceriola (2024) [169]	10	Walking, running (treadmill); optoelectronic	Hip	Sagittal	*ε*ROM	2.8–3.8°	3.1–4.1°
			Knee	Sagittal	*ε*ROM	3.3–3.7°	4.0–5.6°
			Ankle	Sagittal	RMSE	2.1–2.8°	—

## 9. Conclusions

This review provides a comprehensive synthesis of IMU- and vision-based markerless systems for human joint kinematics, with a specific focus on their metrological performance and error propagation. The current literature demonstrates that both technologies have evolved from research prototypes into viable tools for biomechanical assessment; however, their accuracy is not strictly an intrinsic property of the hardware or the neural network but rather a product of the entire measurement and processing chain.

From a metrological perspective, IMU-based systems offer the most consistent reliability for lower-limb sagittal kinematics, where Root Mean Square Errors (RMSE) are typically contained within 3–$6^{\circ }$ under controlled conditions, and their validity is markedly plane-dependent rather than uniform. Accuracy degrades in transverse-plane rotations, with internal/external rotation errors often exceeding 6–$9^{\circ }$ and reaching $12^{\circ }$ over sustained walking and at distal segments such as the ankle. The values quoted above are drawn from level walking and comparable gait tasks, in which transverse-plane rotations are of small amplitude and weakly excited so that a given absolute error represents a large fraction of the range of motion and is particularly sensitive to heading drift. They should not, however, be read as the upper bound of the problem: in tasks that deliberately load the transverse plane, such as the cutting maneuvers of Edwards et al. [177], transverse-plane errors are larger still, reaching $116.8^{\circ }$ for inertial and $56.6^{\circ }$ for markerless capture against a common optoelectronic reference. Part of the difficulty, moreover, lies in the measurand rather than in either technology, since the minimal detectable change for hip rotation is 7.4–$10.2^{\circ }$ even within a reference laboratory [158]. These discrepancies are systematically driven by the accumulation of integration drift, magnetic disturbances, uncompensated soft-tissue artifacts (STAs), and the inherent uncertainty in sensor-to-segment (S2S) calibration methodologies [9,59].

Vision-based markerless systems relocate the error rather than remove it. Being non-invasive, they are not subject to S2S calibration or to the motion of an attached sensor relative to the underlying bone; anatomical localization error is not thereby removed, but it originates in how keypoints are defined and detected, and the dominant bottlenecks become depth ambiguity, motion blur, and spatial occlusion. Current deep learning architectures yield high accuracy for 2D spatiotemporal parameters and sagittal-plane lower-limb angles, with Mean Absolute Errors around 4–$7^{\circ }$. When 3D kinematics are recovered by triangulation or 2D-to-3D lifting, however, accuracy degrades in ways that do not scale predictably with image quality: a correspondence mismatch between views violates the epipolar constraint and displaces the reconstructed joint center, while a lifting network can fail by a wide margin when a joint is occluded or only partly visible. Complex joints and upper-limb rotations frequently exhibit severe deviations—forearm pronation/supination reached $37.4^{\circ }$ within one musculoskeletal pipeline, against $6.8^{\circ }$ for elbow flexion/extension in the same study—reflecting the mismatch between keypoint detection and the rigid-body constraints required by classical Inverse Kinematics (IK) [12,16,57].

Ultimately, neither technology universally replaces optoelectronic capture across all clinical scenarios. IMUs provide robust, high-frequency tracking suitable for unconstrained environments, provided that drift and calibration are strictly controlled. Vision-based systems offer a highly scalable, non-invasive alternative but require rigorous multi-camera geometry to minimize projection errors. Future advancements in human motion analysis must prioritize hybrid data-driven sensor fusion, leveraging markerless global positioning to bound IMU integration drift and inertial data to bridge visual occlusions, alongside adherence to standardized ISB reporting protocols to ensure quantitative reproducibility and clinical interpretability.

## Figures and Tables

**Table 1:** Chronological overview of included reviews by type, first author, year, key topic, and main findings.

Type	1st Auth.	Year	Key Topic	Main Findings
VB	Ke et al. [32]	2013	Video-based HAR	Outlined the main HAR pipeline and application domains.
MIX	Muro-de-la-Herran et al. [39]	2014	Wearable/non-wearable gait analysis	Compared wearable and non-wearable gait analysis options.
MIX	Mooney et al. [40]	2015	Swimming performance analysis	Showed inertial sensing can support detailed swimming analysis.
VB	Simor et al. [41]	2016	Gesture-based games	Reviewed usability methods for gesture-based games.
VB	Colyer et al. [38]	2018	Markerless vision-based MA	Described the shift toward markerless motion analysis.
VB	Zhang et al. [33]	2019	Vision-based action recognition	Summarized vision-based action recognition methods and trends.
VB	Dang et al. [35]	2019	2D pose estimation (DL)	Summarized DL-based 2D pose estimation methods.
VB	Parks et al. [42]	2019	Low-cost video MA in rehab.	Reported mixed validity and low methodological quality.
IMU	Weygers et al. [17]	2020	LL joint kin.	Reviewed methodological requirements for clinical IMU use.
IMU	Pacher et al. [9]	2020	S2S calib. for LB kin.	No standard S2S calibration approach emerged.
IMU	Kobsar et al. [3]	2020	IMU V/R in walking	Strongest evidence for mean spatiotemporal gait measures.
IMU	Zampogna et al. [27]	2020	Wireless Neuro-Balance Sensors	Highlighted broad potential for balance assessment in neurology.
VB	Al-Faris et al. [34]	2020	CV methods for action recognition	Reviewed CV methods from handcrafted to deep learning.
VB	Munea et al. [36]	2020	2D pose estimation models/taxonomy	Organized 2D HPE models into a structured taxonomy.
IMU	Rana et al. [23]	2021	Wearable sensors for real-time sport kin.	Reviewed real-time sensing for performance feedback in sport.
IMU	Prill et al. [25]	2021	Wearable sensors in knee rehab.	Promising reliability, but limited clinical evidence.
VB	Desmarais et al. [12]	2021	3D HPE for markerless MC	Compared 3D HPE methods for markerless MC.
VB	Badiola-Bengoa et al. [43]	2021	Camera-based HPE in sport	Confirmed growing use in sport, but limited dedicated datasets.
VB	Amsaprabhaa et al. [44]	2021	RGB-video gait analysis	Surveyed spatio-temporal RGB-video gait frameworks.
IMU	Lee et al. [18]	2022	Joint kinetics from IMU-MC	Showed IMUs can support kinetics estimation.
IMU	Zeng et al. [20]	2022	IMU V/R in running kin.	Excellent for ST measures; joint-angle RMSE 13–36° in running.
IMU	Schall et al. [21]	2022	Wearable IMUs for objective kin. assess.	Summarized workplace-oriented uses of wearable IMUs.
IMU	Weizman et al. [26]	2022	IMUs in spasticity	Promising use in spasticity, but protocols remain heterogeneous.
IMU	Digo et al. [28]	2022	Wearable inertial sensors in industry	Emphasized HRI, safety, and worker monitoring applications.
IMU	Bo et al. [30]	2022	IMU monitoring in IoHT	Reviewed IMU-enabled assistive monitoring in connected health.
VB	Huamanchahua et al. [45]	2022	Kinect-based motion capture	Reviewed Kinect systems for human movement capture.
IMU	Fang et al. [11]	2023	IMU-to-angle conv. (UL)	Compared conversion methods and accuracy issues for UL angles.
IMU	Kranzinger et al. [22]	2023	IMU motion classification in sport	Mapped ML/DL classification approaches in sport IMU data.
IMU	Gu et al. [24]	2023	IMU-MC for rehab.	Most studies focused on stroke and gait assessment.
MIX	Salisu et al. [29]	2023	MoCap for ergonomics	Showed MoCap is increasingly used for ergonomic risk assessment.
VB	Chen et al. [13]	2023	2D pose estimation survey	Reviewed recent 2D HPE advances and open issues.
VB	Zheng et al. [14]	2023	DL for 2D/3D HPE	Summarized DL solutions for 2D/3D HPE and challenges.
VB	Rybnikár et al. [46]	2023	MoCap for ergonomics eval.	Showed MoCap improves objectivity in ergonomics evaluation.
VB	Sardari et al. [47]	2023	AI for skeleton-based rehab.	Reviewed AI pipelines for skeleton-based rehab evaluation.
IMU	Ghattas et al. [19]	2024	IMU validity in motion tracking	Good agreement in sagittal/frontal planes; large transverse-plane discrepancies.
VB	Neupane et al. [37]	2024	Deep 3D HPE	Reviewed modern deep 3D HPE architectures.
VB	Vun et al. [48]	2024	Vision-based gait in NDDs	Showed promise for gait assessment in NDDs.
IMU	Ashry et al. [31]	2024	TL for IMU-HAR	Highlighted transfer learning as an emerging IMU-HAR strategy.

**Table 2:** Summary of ISB joint coordinate system definitions and the technology-specific measurement challenges of each joint. Quantitative accuracy is reported separately in Section 8.

Joint	Rotational DOFs	Key Challenges (I = IMU; V = Vision)
Ankle	DF/PF Inv/Ev IR/ER	Soft-tissue artifact on foot and shank (I); footwear occlusion (V); few detectable distal keypoints (V)
Knee	Flex/Ext Val/Var IR/ER	Cross-talk in secondary DOFs (I); depth ambiguity in monocular vision (V)
Hip	Flex/Ext Add/Abd IR/ER	Heading drift in rotation (I); pelvic segment instrumentation (I); pelvis region occlusion (V)
Spine	Flex/Ext Axial Rot Lat Bend	Multi-segment approximation only (I, V); for vision, frontal-plane bending is tracked best while sagittal tilt and axial rotation are the limiting DOFs (V)
Shoulder	Flex/Ext Abd/Add IR/ER	Soft-tissue artifact around scapula (I); gimbal lock in YXY Euler sequence (I); self-occlusion during overhead and cross-body movements (V)
Elbow	Flex/Ext Pro/Sup	Forearm sensor misalignment (I); pronation/supination invisible externally (V)
Hand & Wrist	Flex/Ext Rad/Uln Dev	Segments too small for IMU sensors (I); self-occlusion during grasping (V)

*Abbreviations:* DF/PF = dorsiflexion/plantarflexion; Inv/Ev = inversion/eversion; IR/ER = internal/external rotation; Flex/Ext = flexion/extension; Val/Var = valgus/varus; Add/Abd = adduction/abduction; Lat Bend = lateral bending; Rot = rotation; Pro/Sup = pronation/supination; Rad/Uln Dev = radial/ulnar deviation; DOF = degree of freedom.

**Table 3:** Summary of data-driven approaches using IMU data, separated by target output. Joint-angle estimation studies (upper block) and movement-classification studies (lower block) report non-comparable outcome types and are therefore not pooled.

Architecture	Authors	Joints/Target	N. IMUs	Training Data	Reported Performance	Notes
*(a) Joint-angle estimation—outcome: joint angle; metric: RMSE; unit: degrees*						
MLP	Mundt et al., 2021 [74]	Hip, knee, ankle (3D)	5 (sim.)	Synthetic from OMC	nRMSE (range-normalised); sagittal r>0.911	Comparable to LSTM and CNN. The absolute $4.8^{\circ }$ often quoted for this study is from [75]
DL + optimization	Rapp et al., 2021 [79]	Full lower limb (3D)	7 (sim.)	Large synthetic database	RMSE $<\;1.27^{\circ }$ (F/E)	Synthetic training data; no experimental IMU reference
TCN-BiLSTM	Wang et al., 2023 [82]	Hip, knee, ankle	3	Mixed real + synthetic	RMSE 5.21–$9.47^{\circ }$ (sagittal, per joint)	Reduced sensor count; measured (not simulated) signals
*(b) Movement and fault classification—outcome: discrete class label; metric: accuracy or $F_{1}$; unit: dimensionless. These studies do not estimate joint angles and are not comparable with block (a); they are reported here only for completeness of the data-driven landscape.*						
CNN, BLSTM	Anand et al., 2017 [86]	Shot type (wrist-worn)	1	Smartwatch data	$F_{1}$ 78.9–94.6%	Tennis 93.8, squash 94.6, badminton 78.9
SVM, Random Forest	Taborri et al., 2019 [87]	Race-walking fault (knee, ankle)	7	Race-walking IMU data	Accuracy 0.90; best $F_{1}$ 0.89	Loss-of-contact and knee-bent fault detection

*Abbreviations:* MLP = Multilayer Perceptron; BLSTM = Bidirectional Long Short-Term Memory; CNN = Convolutional Neural Network; DL = Deep Learning; TCN = Temporal Convolutional Network; sim. = simulated; OMC = Optical Motion Capture; F/E = flexion/extension; RMSE = root mean square error.

**Table 5:** Comparative analysis of pose estimation models, tasks, and input paradigms.

Model	Task	Paradigm	Input	Temporal	Lift/Triang	IK?
OpenPose [116]	2D	Bottom-up	Image	–	–	No
HigherHRNet [92]	2D	Bottom-up	Image	–	–	No
DEKR [117]	2D	Bottom-up	Image	–	–	No
HRNet [113]	2D	Top-down	Image	–	–	No
ViTPose [114]	2D	Top-down	Image	–	–	No
AlphaPose [100]	2D	Top-down	Image	Yes	–	No
RTMPose (2D) [119]	2D	Top-down	Image	Yes	–	No
RTMO [120]	2D	One-stage	Image	–	–	No
BlazePose [135]	2D/3D	Top-down	Video	Yes	–	No
MoveNet [136]	2D	Single/Multi	Img/Vid	–	–	No
PoseFlow [121]	2D	Bottom-up	Video	Yes	–	No
VideoPose3D [129]	3D	Two-stage	2D Seq	Yes	Lift	No
PoseFormer [137]	3D	Two-stage	2D Seq	Yes	Lift	No
VNect [97]	3D	Single-stage	Image	Yes	–	No
LCR-Net++ [122]	3D	Single-stage	Image	–	–	No
VIBE [138]	Mesh	Mesh Rec.	Video	Yes	–	No
HMR [139]	Mesh	Mesh Rec.	Image	–	–	No
HybrIK [140]	Mesh	Model-based	Image	–	–	Yes
VoxelPose [131]	MV 3D	Volumetric	MV Img	–	–	No
Anipose [133]	MV 3D	Pipeline	MV 2D	Yes	Triang.	No
Pose2Sim [16]	Bio	Pipeline	MV 2D	Yes	Triang.	Yes
OpenCap [15]	Bio	Pipeline	MV Vid	Yes	Triang.	Yes
SynthPose [141]	Bio	Synthetic	Synth 2D	–	–	No

*Note: MV: Multi-view; Seq: Sequence; Lift: Lifting; Triang: Triangulation; Img: Image; Vid: Video.; Bio = Biomechanics-oriented pipeline.*

**Table 6:** Biomechanical validation metrics (kinematics and joint angles).

Metric	Formula
**RMSE** (Root Mean Square Error)	$\mathrm{RMSE}=\sqrt{\frac{1}{N}\sum_{i=1}^{N}{\left({\hat{\theta }}_{i}-\theta_{i}\right)}^{2}}$
**MAE** (Mean Absolute Error)	$\mathrm{MAE}=\frac{1}{N}\sum_{i=1}^{N}\left|{\hat{\theta }}_{i}-\theta_{i}\right|$
**ICC** (Intraclass Correlation)	$\mathrm{ICC}=\frac{\sigma_{b}^{2}}{\sigma_{b}^{2}+\sigma_{w}^{2}}$
**CV** (Coefficient of Variation)	$\mathrm{CV}=\frac{\sigma }{\mu }\times 100$
**Bland–Altman Plot**	$\mathrm{LoA}=\bar{d}\pm 1.96\times {\mathrm{SD}}_{d}$
**Pearson’s *r***	$r=\frac{\sum \left({\hat{\theta }}_{i}-\bar{\hat{\theta }}\right)\left(\theta_{i}-\bar{\theta }\right)}{\sqrt{\sum {\left({\hat{\theta }}_{i}-\bar{\hat{\theta }}\right)}^{2}\sum {\left(\theta_{i}-\bar{\theta }\right)}^{2}}}$
**Mean Difference (MD)**	$\bar{d}=\frac{1}{N}\sum_{i=1}^{N}\left({\hat{\theta }}_{i}-\theta_{i}\right)$

**Table 7:** Computer vision spatial metrics (pose estimation and joint positions).

Metric	Formula/Condition
**PCP** (Percentage of Correct Parts)	$∥{\hat{p}}_{i}-p_{i}{∥}_{2}<0.5\times l_{\mathrm{limb}}$
**PCK** (Percentage of Correct Keypoints)	$∥{\hat{p}}_{i}-p_{i}{∥}_{2}<\alpha \times l_{\mathrm{ref}}$
**PDJ** (Percentage of Detected Joints)	$∥{\hat{p}}_{i}-p_{i}{∥}_{2}<\alpha \times d_{\mathrm{torso}}$
**MPJPE** (Mean Per Joint Pos. Error)	$\mathrm{MPJPE}=\frac{1}{N}\sum_{i=1}^{N}{∥{\hat{p}}_{i}-p_{i}∥}_{2}$
**OKS** (Object Keypoint Similarity)	$\mathrm{OKS}=\frac{\sum_{i}\exp\left(-\frac{d_{i}^{2}}{2s^{2}\kappa_{i}^{2}}\right)\delta \left(v_{i}>0\right)}{\sum_{i}\delta \left(v_{i}>0\right)}$
**PSIM** (Pose SIMilarity)	$\mathrm{PSIM}=f({\mathrm{Sim}}_{\mathrm{pos}},{\mathrm{Sim}}_{\mathrm{struct}},{\mathrm{Sim}}_{\mathrm{int}})$
**TR loss** (Triangulation Residual)	$L_{\mathrm{TR}}=\sum_{v}{∥P_{v}X_{3D}-x_{2D}^{v}∥}_{2}^{2}$

**Table 8:** Examples of clinical applications, the measurement required for each task, the approximate level of accuracy implied by the literature, and technologies that are generally suitable. Values are indicative rather than prescriptive.

Clinical Application	Primary Measurement	Typical Accuracy Needed	Suitable Technologies
Surgical outcome assessment	Joint-angle waveforms and ROM	~2–$3^{\circ }$ (sagittal) [144,157]	Optoelectronic systems; IMUs with validated sensor-to-segment calibration [147]
Surgical planning (transverse plane)	Hip, knee and foot rotation	~7–$10^{\circ }$ MDC; decision thresholds around $15^{\circ }$ [146,158]	Optoelectronic systems; validated multi-camera methods
Injury-risk assessment	Frontal-plane knee kinematics	Effects typically ~$8^{\circ }$ [145]	Optoelectronic or validated multi-camera markerless systems
Rehabilitation follow-up	Range of motion	~2–$6^{\circ }$ [154,155]	Single-camera markerless, smartphone video, IMUs
Functional performance tests	STS, TUG, SPPB outcomes	Agreement with expert ratings [159]	Single-camera markerless or smartphone video
Gait monitoring	Spatiotemporal parameters	High agreement with instrumented walkways [156]	Single-camera markerless or IMUs [20]
Clinical severity scoring	Ordinal clinical scores	Agreement with clinician ratings [148,149]	Consumer video with markerless pose estimation [150,151]
Remote monitoring	Longitudinal within-subject changes	Repeatability more important than accuracy	Consumer video or wearable IMUs

*ROM* = range of motion; *MDC* = minimal detectable change; *STS* = sit-to-stand; *TUG* = timed up-and-go; *SPPB* = short physical performance battery.

**Table 9:** Joint-angle validation studies—IMU-based systems.

Study	Setup	Task	Joint	Plane/DOF	Metric	Value
Lebleu 2020 [51]	7 x-IMU, 3D	Level walking	Knee	Sagittal	RMSE	3.6°
		Stair descent	Knee	Sagittal	RMSE	12.8°
		Obstacle crossing	Knee	Sagittal	RMSE	12.3°
		Stair descent	Hip	Sagittal	RMSE	7.8°
AlBorno 2022 [71]	8 Xsens, 3D	10-min walking	Pelvis, hip, knee, ankle	Sag. + front. (6 DOF, pooled)	RMS diff.	3.0–6.0°
		10-min walking	Hip	Transverse	RMS diff.	12.0°
Potter 2022 [161]	7 IMU, 3D	Normal/atypical gait	Hip	Sagittal	RMS diff. (cl.)	2.2–3.0°
		Normal/atypical gait	Hip	Sagittal	RMS diff. (IK)	5.7–7.2°
		Normal/atypical gait	Hip	Transverse	RMS diff. (cl.)	6.1–12.0°
		Normal/atypical gait	Hip	Transverse	RMS diff. (IK)	6.2–13.5°
McGrath 2022 [53]	magn.-free, 3D	Treadmill gait	Knee	Sagittal	RMSE (rel.)	3.8°
		Treadmill gait	Hip	Sagittal	RMSE (rel.)	3.7°
		Treadmill gait	Hip	Frontal	RMSE (rel.)	4.6°
		Treadmill gait	Hip	Transverse	RMSE (rel.)	6.3°
		Treadmill gait	Knee	Sagittal	RMSE (abs.)	7.9°
		Treadmill gait	Hip	Sagittal	RMSE (abs.)	8.5°
		Treadmill gait	Hip	Frontal	RMSE (abs.)	14.3°
		Treadmill gait	Hip	Transverse	RMSE (abs.)	14.4°
Carcreff 2022 [162]	7 IMU, 3D	Overground walking	Pelvis	Sagittal	RMSE	14.1 ± 2.8°
		Overground walking	Pelvis	Frontal	RMSE	2.2 ± 0.8°
		Overground walking	Pelvis	Transverse	RMSE	2.2 ± 1.1°
		Overground walking	Hip	Sagittal	RMSE	7.8 ± 2.3°
		Overground walking	Hip	Frontal	RMSE	5.1 ± 3.8°
		Overground walking	Hip	Transverse	RMSE	9.6 ± 2.5°
		Overground walking	Knee	Sagittal	RMSE	7.3 ± 3.1°
		Overground walking	Knee	Frontal	RMSE	7.2 ± 3.5°
		Overground walking	Knee	Transverse	RMSE	7.5 ± 3.0°
		Overground walking	Ankle	Sagittal	RMSE	7.2 ± 2.1°
		Overground walking	Foot progr.	Transverse	RMSE	12.2 ± 3.5°
Cerfoglio 2023 [163]	MIMU, 3D	Semi-squat	Thigh (hip)	Sagittal	RMSE (ROM)	3.71°
		Hip flexion	Thigh (hip)	Sagittal	RMSE (ROM)	3.68°
		Trunk lateral bending	Trunk	Frontal	RMSE (ROM)	4.49°
		Knee extension	Shank (knee)	Sagittal	RMSE (ROM)	5.90°
		Hip abduction	Thigh (hip)	Frontal	RMSE (ROM)	6.38°
		Hip extension	Thigh (hip)	Sagittal	RMSE (ROM)	8.47°
		Trunk anterior flexion	Trunk	Sagittal	RMSE (ROM)	14.55°
Wang 2023 [82]	3 IMU, DL	Level walking	Ankle	Sagittal	RMSE	4.4°
		Multi-activity	Hip	Sagittal	RMSE	9.5°
		Multi-activity	Knee	Sagittal	RMSE	8.4°
Rapp 2021 [79]	7 synthetic	Walking, running	Hip	Sagittal	RMSE	<1.3°
		Walking, running	Knee	Sagittal	RMSE	<1.3°
		Walking, running	Ankle	Sagittal	RMSE	<1.3°
Zeng 2022 [20]	meta-analysis	Running	Ankle	Sagittal	RMSE	14.4–19.1°
		Running	Knee	Sagittal	RMSE	13.2–20.0°
		Running	Hip	Sagittal	RMSE	25.1–36.1°
Fang 2023 [11]	review	Upper limb	Shoulder	Humerothoracic	Diff.	∼4.0°
		Upper limb	Shoulder	Glenohumeral	Diff.	≥6.0°
		Upper limb	Shoulder	Scapulothoracic	Diff.	>10.0°
		Upper limb	Elbow	Flex/ext	Diff.	∼2.0°
Fischer 2021 [170]	2 IMU	Dart-throwing	Wrist	Flex/ext	RMSE	8.3 ± 2.5°
		Dart-throwing	Wrist	Radial/ulnar dev.	RMSE	9.9 ± 3.2°
Wirth 2019 [171]	2 IMU	Planar wrist mov.	Wrist	Flex/ext	ROM diff.	10.4 ± 4.2°
		Planar wrist mov.	Wrist	Radial/ulnar dev.	ROM diff.	2.4 ± 1.2°

**Table 10:** Joint-angle validation studies—vision-based (markerless) systems.

Study	Setup	Task	Joint	Plane/DOF	Metric	Value
Stenum 2021 [52]	2D, 1 camera	Overground gait	Hip	Sagittal	MAE	3.7–4.0°
		Overground gait	Knee	Sagittal	MAE	5.1–5.6°
		Overground gait	Ankle	Sagittal	MAE	6.3–7.4°
D’Antonio 2021 [172]	3D, 2 webcams; ref. IMU	Walking, rear + lateral cameras	Hip (R)	Sagittal	Abs. err., peak ext.	1.6 ± 0.3°
		Walking, rear + lateral cameras	Hip (R)	Sagittal	Abs. err., ROM	2.8 ± 1.0°
		Walking, both cameras rear	Hip (R)	Sagittal	Abs. err., peak flex.	2.2 ± 1.2°
		Walking, both cameras rear	Knee (L)	Sagittal	Abs. err., peak flex.	7.3 ± 0.2°
		Running, both cameras rear	Hip (L)	Sagittal	Abs. err., peak ext.	7.2 ± 1.2°
		Running, both cameras rear	Hip (L)	Sagittal	Abs. err., ROM	14.1 ± 1.8°
		All configurations, both tasks	Ankle	Sagittal	Not measurable	—
Gionfrida 2022 [58]	2D, monocular	Hand, radial walking	Fingers (2–5)	Inter-digit splay	RMSE	<9.0°
		Hand, MCP flexion	Fingers (MCP)	Flexion	Error	<11.0°
Thiele 2024 [166]	Theia3D	Weightlifting snatch	Hip	Sagittal	RMSD	22.5 ± 7.0°
		Weightlifting snatch	Hip	Frontal	RMSD	6.6 ± 2.9°
		Weightlifting snatch	Knee	Sagittal	RMSD	8.3 ± 1.9°
		Weightlifting snatch	Knee	Frontal	RMSD	18.2 ± 9.0°
		Weightlifting snatch	Ankle	Sagittal	RMSD	10.0 ± 5.2°
		Weightlifting snatch	Ankle	Frontal	RMSD	9.0 ± 5.7°
Auer 2024 [57]	+ MSK model	Multi-movement	Knee	Sagittal	MAE	4.7°
		Multi-movement	Hip	Sagittal	MAE	7.2°
		Multi-movement	Shoulder	Sagittal	MAE	6.8°
		Multi-movement	Elbow	Flex/ext	MAE	6.8°
		Multi-movement	Hand	Flexion	MAE	15.0°
		Multi-movement	Elbow	Pro/supination	MAE	37.4°
Uhlrich 2023 [15]	3D, multi-view	Gait, squat, STS, drop jump (range across the 4 tasks)	Pelvis	Tilt (sag.)	MAE	4.6–8.7°
			Pelvis	List (front.)	MAE	1.7–2.3°
			Pelvis	Rotation (trans.)	MAE	2.4–3.5°
			Hip	Flexion (sag.)	MAE	5.2–7.1°
			Hip	Adduction (front.)	MAE	2.6–2.8°
			Hip	Rotation (trans.)	MAE	3.2–4.4°
			Knee	Sagittal	MAE	2.9–4.9°
			Ankle	Sagittal	MAE	3.3–8.5°
			Subtalar	Inv./eversion	MAE	5.1–6.5°
			Lumbar	Extension (sag.)	MAE	6.6–10.3°
			Lumbar	Bending (front.)	MAE	2.0–3.9°
			Lumbar	Rotation (trans.)	MAE	3.1–5.6°
Ceriola 2024 [169]	3D, RTMPose-FT	Walking 3.5 km/h	Knee	Sagittal	*ε*ROM	4.0 ± 3.7°
	3D, RTMPose-FT	Running 7.0 km/h	Hip	Sagittal	*ε*ROM	3.1 ± 1.8°
	3D, MediaPipe	Walking 3.5 km/h	Hip	Sagittal	*ε*ROM	3.5 ± 1.9°
	3D, MediaPipe	Running 7.0 km/h	Knee	Sagittal	*ε*ROM	4.8 ± 4.3°
Wishaupt 2024 [173]	Theia3D	Gait (cerebral palsy)	Trunk	Tilt (sagittal)	RMSE	9.2–11.8°
		Gait (cerebral palsy)	Trunk	Lateroflexion	MAD	1.5–3.0°
		Gait (cerebral palsy)	Hip	Sagittal	RMSE	5.0–8.0°
		Gait (cerebral palsy)	Hip	Transverse	RMSE	9.0–13.0°
Croci 2025 [174]	Theia3D	Full arm ROM	Shoulder	Abduction	RMSD	<6.0°
		Full arm ROM	Shoulder	Axial rotation	RMSD	15.0–22.0°
Lahkar 2022 [175]	Theia3D	Boxing	Shoulder	Abd/adduction	RMSD	6.3–6.6°
		Boxing	Shoulder	Flex/ext	RMSD	7.3–10.0°
		Boxing	Shoulder	Axial rotation	RMSD	8.1–12.0°

**Table 11:** Joint- and landmark-*position* validation studies (unit: mm); not comparable with Table 9 and Table 10.

Study	Setup	Task	Target, Metric	Value
Zago 2020 [164]	3D, stereo	Overground gait	13 landmarks, traj. error	21–64 mm
		Overground gait	Single landmark, traj. error	16–103 mm
Thiele 2024 [166]	Theia3D	Weightlifting snatch	LL joint centres, mean diff.	47 ± 12 mm
		Weightlifting snatch	UL joint centres, mean diff.	57 ± 15 mm
D’Antonio 2021 [172]	3D, 2 webcams	Treadmill gait, both cameras rear	Knee centres (L/R), RMSE	24–33 mm
		Treadmill gait, both cameras rear	Hip centres (L/R), RMSE	15–16 mm

**Table 12:** Spatiotemporal gait validation studies (unit: s, m, or steps/min); not comparable with the tables above.

Study	Setup	Parameter	Metric	Value
Stenum 2021 [52]	MK, 2D	Step length (per step)	MAE	0.049 m
		Step length (per subject)	MAE	0.018 m
Zago 2020 [164]	MK, stereo	Swing time	RMSE	0.02 s
		Stance time	RMSE	0.05 s
Potter 2022 [161]	IMU, 7 sensors	Stride length, step width	RMS diff.	<0.13 m
Lonini 2022 [156]	MK (DLC), 2D	Cadence	Mean diff.	−0.25±3.9 steps/min
		Walking speed	Mean diff.	−0.02±0.11 m/s
Zeng 2022 [20]	IMU, meta-anal.	Stride length	ICC	0.937
		Step frequency	ICC	0.926

## Data Availability

No new data were created or analyzed in this study. Data sharing is not applicable to this article.
